# Lanthanide‐Doped Upconversion Nanoparticles: Emerging Intelligent Light‐Activated Drug Delivery Systems

**DOI:** 10.1002/advs.201500437

**Published:** 2016-03-15

**Authors:** Ali Bagheri, Hamidreza Arandiyan, Cyrille Boyer, May Lim

**Affiliations:** ^1^ School of Chemical EngineeringThe University of New South WalesSydneyNSW2052Australia; ^2^ Center for Advanced Macromolecular Design (CAMD)and Australian Center for NanoMedicine (ACN)School of Chemical EngineeringUNSW AustraliaSydneyNSW2052Australia

**Keywords:** controllable drug delivery, near‐infrared, photocleavage, photoswitching, upconversion nanoparticles

## Abstract

The development of drug delivery systems (DDSs) using near infrared (NIR) light and upconversion nanoparticles (UCNPs) has generated intensive interest over the past five years. These NIR‐initiated DDSs not only offer a high degree of spatial and temporal determination of therapeutic release but also provide precise control over the released dosage. Furthermore, these nanoplatforms confer several advantages over conventional light‐based DDSs—NIR offers better tissue penetration depth and a reduced risk of cellular photo‐damage caused by exposure to light at high‐energy wavelengths (e.g., ultraviolet light, <400 nm). The development of DDSs that can be activated by low intensity NIR illumination is highly desirable to avoid exposing living tissues to excessive heat that can limit the in vivo application of these DDSs. This encompasses research in three directions: (i) enhancing the quantum yield of the UCNPs; (ii) incorporation of photo‐responsive materials with red‐shifted absorptions into the UCNPs; and (iii) tuning the UCNPs excitation wavelength. This review focuses on recent advances in the development of NIR‐initiated DDS, with emphasis on the use of photo‐responsive compounds and polymeric materials conjugated onto UCNPs. The challenges that limit UCNPs clinical applications, alongside with the aforementioned techniques that have emerged to overcome these limitations, are highlighted.

## 1 Introduction

The development of drug delivery platforms that can deliver a therapeutic payload in a controlled manner to an internal target site is one of the most active research areas in the field of nanomedicine. Particularly, stimuli‐triggered drug delivery systems (DDSs) that can release the bioactive compounds with precise control over timing, site, and dosage of the released payload have received considerable attention in the past six years.^1^, ^2^, ^3^

To date, a number of stimuli have been employed in novel DDSs to trigger the release of payloads, comprising temperature, pH, magnetic field and light. Of these, light has attracted special attention due to its intrinsic features of being spatially and temporally tunable such that precise “on‐command” release of a wide range of therapeutic compounds, including but not limited to photosensitizers, chemotherapeutic drugs, genes and proteins, can be triggered remotely.^4^, ^5^, ^6^, ^7^, ^8^, ^9^ Furthermore, as opposed to other triggers such as pH and temperature, light in the visible to NIR range was no adverse effect on the physiological function of living tissue (under a moderate light intensity), which is a vital prerequisite for bio‐medical applications. The ability to control when and where a drug is released via photoactivation enhances the local effective drug accumulation alongside minimizing the drug's toxic adverse effects, resulting in improved drug efficacy and patient comfort.^10^, ^11^ Nonetheless, most of the light‐triggered DDSs developed in recent years have certain limitations that hamper their practical applications. Notably, most of the photosensitive compounds employed in DDSs required high‐energy wavelengths (UV or visible light) and high intensity to be activated; both UV and visible light have poor tissue penetration depth, and prolonged exposure to UV can cause severe cellular photodamage.^12^, ^13^, ^14^

To facilitate lower phototoxicity and increase the depth of tissue penetration, NIR‐to‐UV/visible up‐conversion nanoparticles (UCNPs) have emerged as potential remote‐controlled nanotransducers to generate in situ the unfavorable UV/visible source in order to activate the photoreaction of photosensitive molecules that form a part of the DDS.^15^, ^16^ Indeed, UCNPs can convert low‐energy and deeply penetrating NIR to high‐energy radiation, such as UV/visible/NIR spectral range through a phenomenon known as photon upconversion. Several comprehensive reviews related to upconversion principles, controllable synthesis of monotonic UCNPs and also geometry and electronic structure of lanthanide (Ln^3+^) in the host lattice have been published.^17^, ^18^, ^19^ The applications of UCNPs such as in vitro and in vivo bioimaging, photodynamic therapy, photothermal therapy, deep 3D bioimaging as well as solar energy harvesting applications and security encoding applications, are numerous and would be beyond the scope of this review, therefore we can only refer to other reviews.^20^, ^21^, ^22^, ^23^, ^24^, ^25^, ^26^, ^27^ Herein, the focus is on the underlying principles behind incorporating UCNPs into light‐initiated DDSs where the nanoparticles act as an in situ source of up‐converted UV or visible light that can be used to trigger drug release through photodegradation of photoresponsive moieties that are incorporated into the DDSs.^28^, ^29^, ^30^, ^31^ NIR light‐initiated nanoplatforms are significantly growing as prospective candidates to revolutionize light‐activated carriers with the potential to overcome the drawbacks associated with conventional light‐activated DDSs.^32^ The use of UCNPs in light‐activated DDSs requires modification of the UCNPs surface. This is because most of the UCNPs produced by existing synthesis techniques are hydrophobic in nature, which restricts their usage in biomedical application. The most rational approach to reducing the hydrophobicity, and therefore improving the compatibility of UCNPs‐based DDSs with biological systems, is through the incorporation of water‐soluble polymers into their design. In addition to improved solubility in aqueous media, modification with polymer also offers UCNPs the surface functionality that can be used for further bioconjugation as well as photoresponsivity. Light‐sensitive functionalities can be introduced into the polymer network (covalently bound within the polymer backbone or attached as side groups) or through composite systems in which a light‐responsive group is suspended within the network, though not covalently attached.^33^, ^34^, ^35^

Another issue, which remains to be solved, is the low absorption of Yb^3+^ doped UCNPsat the 980 nm wavelength and the high absorption of light at around the 980 nm wavelength by water and biological system, which can cause a deleterious excessive heat effect. To ameliorate this shortcoming research has been directed towards the development of new generations of UCNPs with more biocompatibility. These endeavors encompass three directions: (i) Improving the quantum yield of UCNPs through core and shell structure; (ii) incorporation of photoresponsive materials into UCNPs that can be activated with visible‐light emitted from UCNPs under ultralow‐intensity 980 nm light source; and (iii) design of next generation neodymium (Nd^3+^)‐doped UCNPs, which can be excited by a 800 nm light source with higher tissue penetration depth and minimal heat issues due to low absorption of water at this wavelength alongside with the development of a new family of dye‐sensitized UCNPs with a broadened absorption range.^36^, ^37^, ^38^

**Figure 1:**
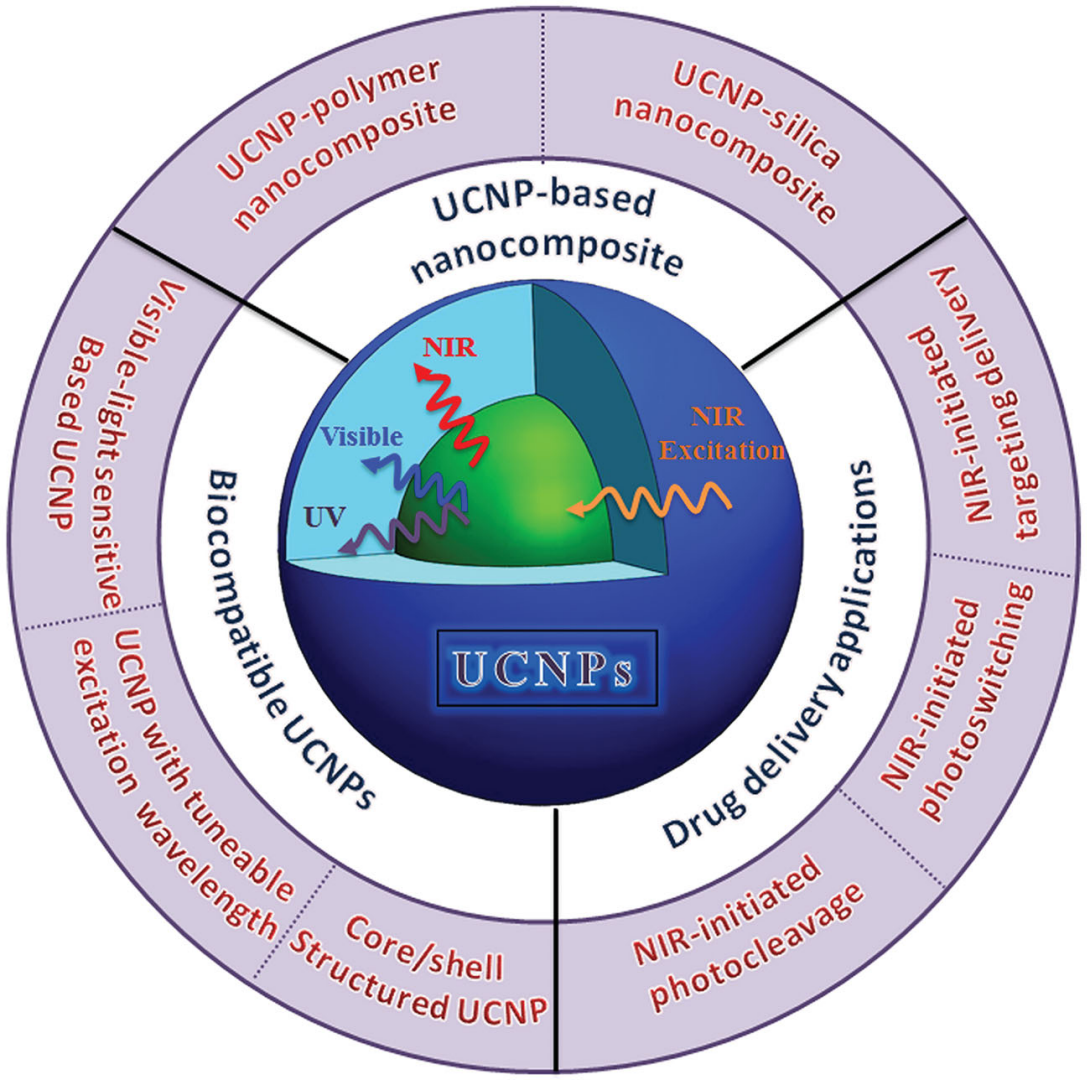
Schematic illustration of the main topics covered in this review.

With regards to the recent upsurge of investigations on NIR‐initiated DDSs, a comprehensive review is greatly demanded based on the current literature related to NIR controlled drug delivery. An overview of recent advances in the field of UCNP‐based light‐activated DDSs and highlights of new development of UCNPs/polymer hybrids is provided herein. Challenges that limit UCNPs potential use in biomedical applications together with prospective solutions are also addressed. Finally, this article spotlights recent progress on the tuning of the excitation wavelength towards 800 nm that will pave the way for widespread growth of new generations of UCNPs with greater biocompatibility, minimal overheating effects and deeper penetration depth. The main themes of this review are represented in **Figure** **1**.

## 2 Upconverted Mechanism of UCNPs and Construction of UCNPs

Based on the principle of Stokes law, emitted photons are at a lower energy (higher wavelength) compared with excitation photons and for all fluorescence/phosphorescence light emitters with involvement of only one excited ion, this principle is valid. However, it has been shown that Ln ions when doped in solids may deviate from this process, emitting higher energy photons upon excitation, in what is known as anti‐Stokes emissions or upconversion (UC) processes.^39^, ^40^, ^41^ The UC route has been widely investigated in recent years and proven to be a promising approach for emitting UV/visible/NIR light in response to NIR light illumination. The UC phenomenon requires the successive absorption of two or more photons to facilitate enough energy for the occurrence of the upconverted emission. To date, various types of UC mechanisms of Ln have been recognized, which might be involved in this process either alone or in combination:^9^, ^42^, ^43^ (i) excited‐state absorption (ESA); (ii) energy transfer upconversion (ETU); (iii) cross relaxation; and (iv) cooperative sensitization upconversion (CSU), and there is a large body of literature describing these principal in details.^40^, ^44^, ^45^ The ETU route is of practical importance for UCNPs studies, since the most efficient UCNPs thus far have exploited this pathway for enhanced excitation and highly efficient upconverted emissions.^46^

Ln‐doped UCNPs that exploited the ETU pathways typically consist of three essential constituents: a host matrix, activators and sensitizers.^27^, ^47^ The sensitizer and activators are two neighboring ions with different energy levels that absorb a photon of identical energy, thus filling the metastable level E_1_.^40^ A non‐radiative energy transfer promotes the activator to a higher emitting state E_2_ whereas the sensitizer relaxes back to its ground state G.^40^ Another pathway has been proposed for the ETU process, in which the sensitizer absorbs a pump photon and excited to its metastable level E_1_. This harvested energy subsequently promotes activator to E_1_ level and ultimately the upper emitting state E_2_ level when the sensitizer relaxes back to ground‐state G twice.^32^

Highly efficient Ln‐doped UCNPs needs a rational tuning between crystalline host, doping ions and dopant concentration.^36^ The selection of the crystalline host matrix is therefore critical due to its influence on the luminescence yield and the emission intensity ratios of diverse energy transitions.^44^ The main criteria the host matrix needs to fulfill are the ability to accommodate the Ln dopant ions and having low phonon energies. In addition, the host matrix should also produce homogeneous doping, high chemical stability, and show minimal lattice stress and non‐radiative energy losses.^40^ Fluoride‐based lattices, such as LaF_4_, YF_4_, NaYF_4_ and BaYF_4_, have been employed extensively as host matrices as they meet many of the above criteria.^17^ Up to early 2012, NaYF_4_ was recognized as the most efficient host lattice for intense visible emissions. Hexagonal phase NaYF_4_ (β‐NaYF_4_) nanocrystals were preferable over the cubic form (α‐NaYF_4_) due to greater upconverted emissions of the former.^5^, ^47^, ^48^ However, recently in 2012, Li's group demonstrated that the cubic NaLuF_4_ host matrix (sub‐20 nm) doped with Er^3+^/Tm^3+^ ions has substantially stronger UC luminescent intensities (10‐times enhancement) in comparison with that of 20 nm β‐NaYF_4_ host‐based UCNPs.^49^

Trivalent Yb^3+^ and Nd^3+^ ions are the most extensively used sensitizers in UCNPs that are activated by 980 nm and 800 nm light, respectively, due to their larger cross section absorption around these NIR wavelengths when compared with that of other lanthanides.^50^, ^51^ Yb^3+^ ions are normally embedded in relatively high doping concentration (20%–30%) due to their only one excited level (^2^F_5/2_) in order to provide efficient energy transfer to neighboring ions.^52^ In contrast, due to the different energy levels of Nd^3+^ and possible deleterious cross relaxation effects, the optimal doping concentration of Nd^3+^ into the UCNPs structure is more challenging, which will be discussed in details in section 6.

**Figure 2:**
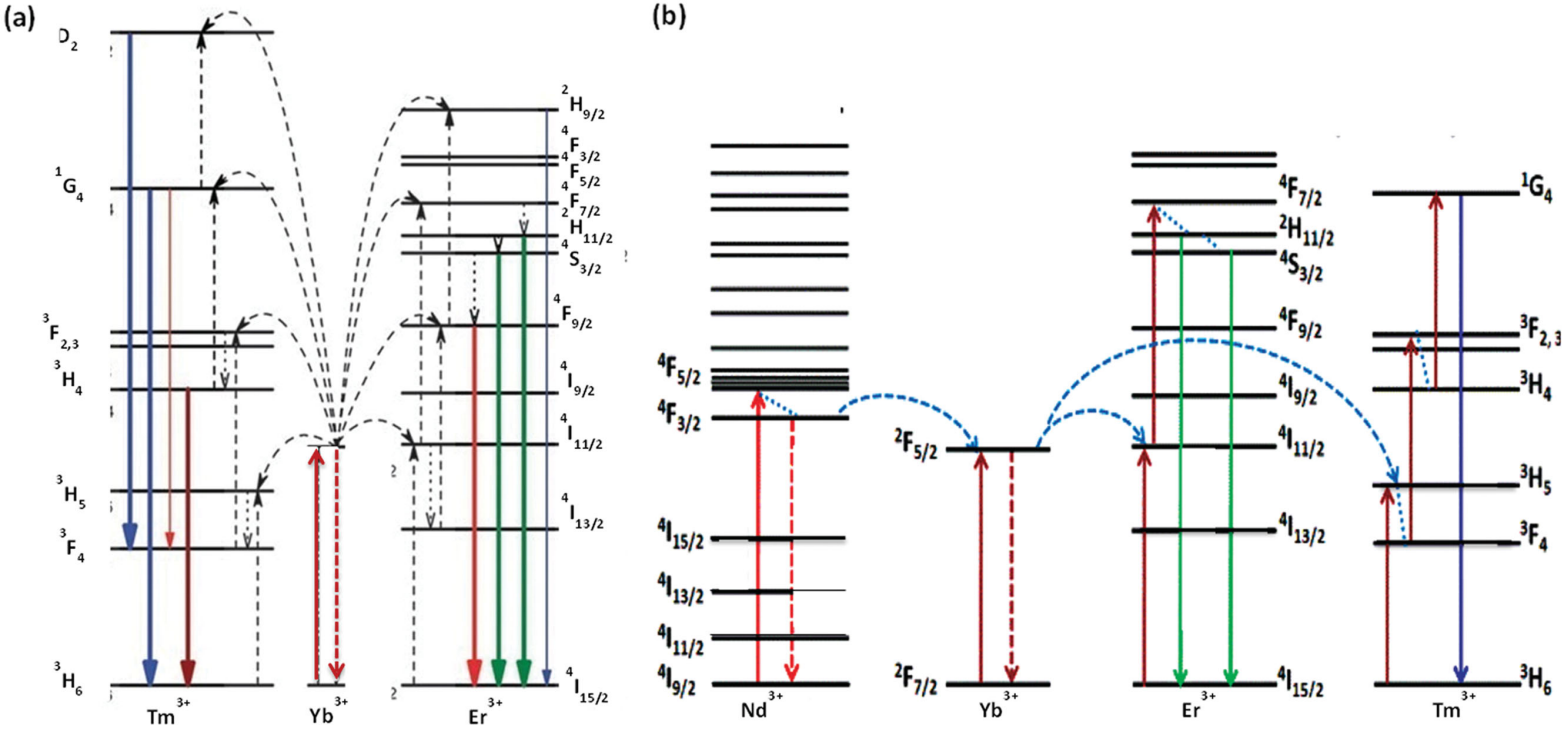
Proposed energy transfer mechanisms showing the UC processes in a) Er^3+^, Tm^3+^, and Yb^3+^ doped UCNPs under 980‐nm diode laser excitation. Reproduced with permission.^40^ Copyright 2009, Royal Society of Chemistry b) Nd^3+^, Yb^3+^, and Er^3+^/Tm^3+^ ions upon 800 nm excitation. Reproduced with permission.^167^

Generation of efficient UC emission requires close energy difference between each excited level and its lower‐lying energy level in order to enable photon absorption and energy transmission steps involved in ETU processes. Therefore, lanthanides with ladder‐like arranged energy levels such as Er^3+^, Tm^3+^, and Ho^3+^ are frequently used as activators with low doping ratios (<2%) to avoid detrimental cross‐relaxation issues.^53^ Composition mapping studies carried out by Yin et al. have clearly proven that an improper sensitizer/activators concentration ratio will lead to concentration‐related cross‐relaxation/quenching or disable energy transfer.^52^ In particular, it should be emphasized that the anti‐Stokes emission efficiency of ETU route is strongly dependent on the distance between ions, which is determined by the concentrations of ion dopants.^40^, ^52^, ^54^, ^55^, ^56^ To date, monodispersed core–shell UCNPs comprising of NaYF_4_ nanocrystals doped with lanthanides, such as Tm^3+^ and Yb^3+^ (NaYF_4_:TmYb) have been reported as promising candidates for the upconversion process.^11^, ^57^, ^58^ The proposed ETU mechanism of the most thoroughly investigated UCNPs with NaYF_4_: Yb^3+^/Er^3+^ (Tm^3+^) structure and also highly efficient Nd^3+^‐sensitized UCNPs are illustrated in **Figure** **2**a,b respectively and explained in detail in the literature.^59^, ^60^, ^61^

### 2.1 Principle behind Adaptation of UCNPs for Use in Drug Delivery Systems

The integration of UCNPs into NIR‐initiated DDSs is driven by the high degree of control over the location, timing and dosage of therapeutic release offered by the use of NIR light triggers.^11^, ^62^, ^63^, ^64^ The use of UCNPs in DDSs entails several technical requirements to be met. First, pure‐phase, uniform, monodisperse UCNPs that do not exhibit toxicity towards cells and to the body have to be fabricated in a high‐quality manner. Next, surface modification of the UCNPs is essential in order to transfer the nanoparticles from an organic into an aqueous phase for biological applications. Then, appropriate materials are required to provide coupling chemistry or a pore structure in order to incorporate the drug into the UCNPs by either physical absorption or covalent bindings. The drug coupling material may have light responsive properties and absorption that overlaps with that of the anti‐Stokes emissions generated from UCNPs is required. Finally, surface engineering of the UCNPs to provide functional groups that can improve the targeting of diseased tissue and thus enhance the efficacy of drug via targeted therapy, particularly in cancer therapy.^65^, ^66^

## 3 Synthesis of UCNPs‐based Composites for Use in Drug Delivery Applications

To date, researchers have established a diverse range of synthetic routes in order to provide UCNPs with well‐controlled particles size and morphology, compositions and upconverted emission bands.^52^, ^67^, ^68^, ^69^ Preparation techniques such as thermal decomposition, hydrothermal/solvothermal method, ionic liquids‐based synthesis, and combustion have been widely reported and reviewed in the literature.^9^, ^27^, ^40^, ^70^, ^71^ Of these, wet‐chemical approaches such as thermal decomposition and hydrothermal/solvothermal are leading synthetic tactics in the biological research area due to their potential for producing nanoparticles with narrow size distribution and high dispersibility. More recently, strategies to integrate other functional moieties with the synthesized UCNPs to form a DDSs platform have undergone rapid development. Nanocarriers that consist of UCNPs integrated with other materials can be broadly separated into two main categories (i) UCNP‐silica nanocomposites; and (ii) UCNPs‐polymer nanocomposites, which have witnessed a rapid growth for drug delivery applications. The following two sections describe these two approaches.

### 3.1 Synthesis of UCNP–Silica Composites

Coating with silica is one of the most common techniques for the surface engineering of UCNPs. The silica exhibits low cytotoxicity and excellent chemical stability as well as the potential to functionalize the inner pore system and/or the external particle surface. In terms of biocompatibility, silica is accepted as “generally recognized as safe” (GRAS) by the United States of America ‐ Food and Drug Administration (FDA).^72^, ^73^, ^74^ For these reasons, recently, substantial efforts have been focused on integrating UCNPs with silica and/or mesoporous silica, by manipulating the architect design and synthetic approaches, for the development of novel drug delivery vehicles. A substantial body of literature demonstrating this principal have already been published.^75^, ^76^, ^77^, ^78^, ^79^ Recently, Liu et al. has thoroughly reviewed the developments in the area of UCNPs@silica structures, including those coated with dense silica, mesoporous silica, or hollow mesoporous silica.^80^ Depending on the polarity of the ligands on the outer surface of UCNPs, different methods can be employed for silica coating. In first approach, often refered to as the Stöber method, tetraethyl orthosilicate undergoes a series of condensation reactions that convert the silica precursor molecule into a mineral‐like deposition on the surface of nanoparticles via the formation of Si–O–Si linkages.^81^ The Stöber method is the most common technique for coating UCNPs with silica due to its simplicity, robustness and versatility. Another route is the water‐in‐oil reverse microemulsion method where hydrophobic capping ligands are used to promote the deposition of uniform and monodisperse silica onto he UCNPs.^82^ This method exploits chemical reactions in nano‐sized hydrophilic cavities generated by a homogeneous mixture of ammonia, cyclohexane, surfactants (Igepal CO‐520, TritonX‐100, cetyltrimethylammonium bromide) and tetraethyl orthosilicate.

**Figure 3:**
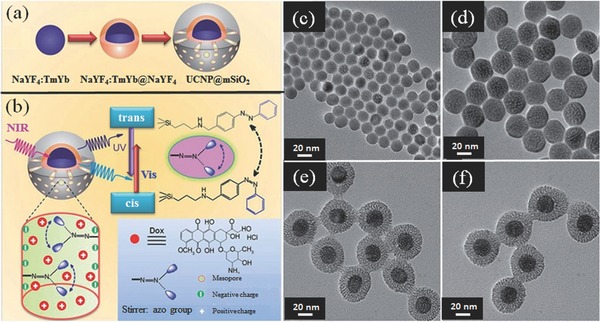
a) Synthetic procedure for upconverting nanoparticles coated with a mesoporous silica outer layer. b) NIR light‐triggered DOX release by making use of the upconversion property of UCNPs and trans–cis photoisomerization of azo molecules grafted in the mesoporous network of a mSiO_2_ layer. Transmission electron microscopy (TEM) images of c) NaYF_4_: Tm, Yb, d) NaYF_4_: Tm, Yb@NaYF_4_, e) NaYF_4_:Tm, Yb@NaYF_4_@mSiO_2_, andf) NaYF_4_:Tm, Yb@NaYF_4_@mSiO_2_‐azo. Reproduced with permission.^86^

Once the surface of the UCNPs is covered with silica, functional groups such as amines, carboxyl, or thiols can easily be introduced via condensation of functional trimethoxysilane; the functional groups can then be used to attach targeting agents, polymers or photosensitive molecules to the UCNPs.^78^, ^83^, ^84^, ^85^ Additionally, the silica surface usually has a porous structure which provides a large surface area for loading drugs and other bioactive compounds. For example, Shi's group demonstrated the first direct NIR‐initiated controllable chemotherapeutic doxorubicin (DOX) release based on silica coated NaYF_4_:TmYb@NaYF_4_ UCNPs modified by photo‐switchable azobenzene molecules for drug release and transactivating transcriptional activator (TAT) peptide to enhance the cellular uptake. The DOX molecules (positively charged) are strongly bound to the negatively charged silica surface (zeta potential: –40.6 mV) through the formation of hydrogen bonds and electrostatic interactions with the surface silanol groups. The controlled release of DOX is triggered by the reversible photoisomerization of azobenzene molecules after exposition of upconverted UV and visible light emissions generated from UCNPs under NIR light (**Figure** **3**).^86^ Besides from dense silica coating, rattle‐structured UCNPs@hollow mesoporous silica strategy has emerged as a promising approach to fabricate a versatile nanoplatform.^76^, ^80^ In this context, Li et al. reported innovative architecture design of UCNPs functionalized eccentric single‐hole mesoporous silica nanocages to independently control the dual‐drug delivery in respond to NIR light and heat.^75^ These nanoarchitectures were synthesized through three stages; First, coating a dense silica layer on the outer surface of UCNPs (NaGdF_4_:Yb, Tm@NaGdF_4_). Second, using UCNPs@SiO_2_ as the initial seed, periodic mesoporous organosilica was coated on the surface of the seed to form the eccentric core@shell nanocomposites. Then, etching (partial dissolution of the interior of nanoparticles) approach was employed to create the eccentric hollow structured nanocomposite. Finally, the single hole was created on the hollow shells by etching the as‐synthesized hollow structured nanoplatforms. The outer surface of the nanoparticles with an open hole (≈25 nm), which acted as a storage space and passage for large bioactive compounds (bovine serum albumin; 21 × 4 × 14 nm^3^), was modified by heat‐sensitive phase change materials (1‐tetradecanol). Meanwhile, the mesoporous shell channels (2–10 nm), which accommodated small DOX molecules (<1 nm^3^), were modified by light responsive azobenzene molecules. In response to NIR light illumination and heat treatment (≈40 °C) dual‐delivery of the bioactive compounds with different size were well‐controlled independently.^75^

### 3.2 Synthesis of UCNP‐Polymer Composite

For many applications, particularly in the drug delivery field, UCNPs are required to be stable in water with a nanorange size (<200 nm) and narrow size distribution, biocompatibility and the feasibility for surface functionalization. Up to the present, UCNPs are typically fabricated using wet‐chemical techniques, which result in the production of size‐ and shape‐controlled UCNPs with high crystallinity and high upconverted emission efficiency. Due to the shortcomings of existing synthesis techniques, UCNPs are hydrophobic in nature owing to the usage of capping surfactants (e.g., oleic acid (OA)), which restricts their usage in the biomedical application.^87^ To this end, continuing efforts have been directed toward investigating a variety of surface modification strategies to transfer hydrophobic UCNPs into aqueous phase. Furthermore, the polymer layer confers increased circulation time of nanoparticles in the bloodstream and attenuates nonspecific uptake by cells in the reticuloendothelial system.^11^, ^37^, ^88^ Following this concept, many diverse types of polymers have been employed to render the UCNPs water‐dispersible and further providing bioactive chemical moieties for bioconjugation and biofunctionalizations.^17^, ^40^, ^89^ More importantly, polymers can be designed to be light‐responsive, which allows the remote release of therapeutic agents. **Table** **1** lists polymers and their applications that have been explored by different research groups in the field of UCNPs/polymer DDSs. The focus of the following sections is centered on polymers and their representative strategies for interactions with UCNPs to modify them for biomedical applications.

**Table 1:** Polymer and their applications in the field of UCNPs/polymer DDSs

UCNP platform	Incorporated Polymer	Effect of Polymer	Therapeutic cargo	Application	Ref.
NaYF_4_:Yb/Tm@NaYF_4_‐ −hydrogel	PVP/Hydrogel	Endow water solubility/hide bioactives inside the hydrogel matrix	Biomacromolecules	On demand bioactive release	65
NaYF_4_:Yb,Er	PEG	Provide bioconjucation of targeting ligands	DOX	Targeted cancer cell imaging and therapy	87
		Endow water solubility and biocompatibility			
Hollow mesoporous structured α‐NaYF_4_:Yb,Er	PEI	Endow water solubility and biocompatibility	DOX	Cell imaging and targeted anti‐cancer drug delivery	97
		Provide bioconjucation of targeting ligand			
		Stability enhancement of precursors			
NaY/GdF_4_:Yb,Er	PEG/PEI/OA‐PAA	Endow water solubility and stability in various physiological solutions	Antigen (OVA)	Immunotherapy	100
		Endow highly positively charged nanoparticles			
NaYF_4_:Yb,Er@NaYF4@SiO_2_@m‐SiO_2_	PAH	Remain UCNPs carrying the NO photochemical precursors intact	NO	Photochemical delivery of bioregulatory molecules	121
NaYF_4_:Yb,Er@SiO_2_	PVP	Control the size and stability of the nanocrystals		Fluorescent probes in cell imaging	144
		Render UCNPs dispersible in organic solvents and water			

#### 3.2.1 Ligand Exchange

Most studies so far focus on hydrophilic polymers, such as polyvinyl pyrrolidine (PVP),^90^ poly(amidoamine) (PAMAM),^91^ multidentate carboxylic groups containing (–COOH) polymers,^92^, ^93^ polyethylenimine (PEI),^94^ polyacrylic acid (PAA),^95^ and multidentate thiolate‐grafting (–SH) polymers.^96^ These polymers owing to their functional groups are excellent candidates to displace the hydrophobic ligands on the surface of nanoparticles and provide a hydrophilic surface and/or chemical groups for bioconjugation in biomedical applications. For instance, in 2009, Capobianco and co‐workers employed PAA polymer to transfer the OA‐coated UCNPs (NaGdF_4_:Ho^3+/^Yb^3+^) to aqueous phase via two the ligand exchange technique. Following the complete ligand exchange with PAA, transparent aqueous solution of UCNPs was produced with significant stability enhancement (greater than three months) with no noticeable decrease in upconversion luminescence intensity.^95^ Interestingly, in the same study, an oxidation approach was conducted for conversion of hydrophobic UCNPs into water‐soluble nanoparticles using oxidizing agent (permanganate/periodate). The oxidation process led to the exploitation of carbon‐carbon double bonds, which can be transformed into a carboxylic acid group. The COOH moiety endows UCNPs with better water solubility and can be employed for subsequent biofunctionalizations. Based on their results, oxidation beyond 2 hours resulted less water dispersibility of UCNPs as well as weak upconversion luminescence intensity. This strategy is appropriate for confined number of ligands with unsaturated carbon‐carbon bonds. This is a potential limiting issue which hinders the usage of this technique.

Furthermore, Lin's group reported that PEI ligands played a critical double role towards the synthesis of hollow mesoporous α‐NaYF_4_:Yb^3+^, Er^3+^ UCNPs; On the one hand, PEI ligands allow the production of water‐soluble and biocompatible UCNPs while providing free functional amine groups on the surface of nanoparticles for further functionalization with folic acid (FA). On the other hand, PEI ligands can effectively protect the upconverting nanoparticles against acid media, which results in stability enhancement.^97^ Another outstanding example was reported by Lin's group, who stabilized OA‐capped core−shell structured NaYF_4_:Yb^3+^/Tm^3+^@NaGdF_4_:Yb^3+^ nanoparticles using PEI as functional ligands to transfer UCNPs into aqueous phase. Lin and co‐workers employed these water‐soluble UCNPs to combine trimodality imaging (magnetic resonance/upconversion luminescence/computer tomography) properties for cancer diagnosis together with NIR‐initiated platinum prodrug delivery for cancer therapy purposes. It should be pointed out that in their structure; a monolayer of PEG coated on the surface of UCNPs is introduced in order to gain nanoparticle stability and to reduce the immunogenicity and antigenicity from the host's immune system.^98^ In addition to the examples mentioned above, Branda and co‐workers used another water‐ compatible polymer, PVP, to make the NaYF_4_:Yb/Tm@NaYF_4_ nanoparticles dispersible in water through replacing the hydrophobic oleate ligands in order to incorporate the UCNPs into the hydrophilic hydrogel to innovate NIR‐controllable materials for uncaging and thus controlled release of bioactive compounds.^65^

#### 3.2.2 Ligands Attraction

To date, tremendous efforts have been devoted to render the hydrophobic nanoparticles water solubile by employing the amphiphilic polymer attachment on the surface of nanoparticles, which is known as ligand attraction approach.^46^ Based on this strategy, in contrast to the ligand exchange technique (that original ligands on UCNPs is replaced by functional hydrophilic polymers), van der Waals forces result in the interaction of hydrophobic segment of the amphiphilic polymer with the hydrophobic ligands capped on the outer layer of nanoparticles. Consequently, the linkage between the hydrophilic segment of the amphiphilic polymer and aqueous solution results in water solubility of the nanoparticles. Some of the most frequently employed amphiphilic polymers encompass polyethylene glycol (PEG)‐lipid,^87^ poly((ethylene glycol)‐*block*‐lactic acid) (PEG‐*b*‐PLA),^99^ octylaimne‐block‐poly(acrylic acid) (OA‐PAA),^100^ octylamine‐*block*‐poly(acrylic acid)‐*block*‐polyethylene glycol (OA–PAA–PEG) copolymer,^101^ poly(styrene)‐*block*‐(allyl alcohol) (PS‐b‐PAA),^102^ poly(ethylene glycol)‐*block*‐poly(carprolactone) (PEG‐b‐PCL)^103^ and TWEEN.^104^ For instance, Liu's group has reported the coating of NaYF_4_: Yb, Er multifunctional system with amphiphilic PEG polymer for targeted delivery of chemotherapy drugs and cell imaging. In their design, UCNPs capped with OA were encapsulated by PEG grafted amphiphilic polymer to produce a “hydrophobic pocket” with the feasibility of physically absorbance of lipophilic drugs (DOX) into the pocket by hydrophobic interaction.^87^

Following this study, Zhao's research group employed TWEEN (polysorbate surfactant) to innovate a facile and low‐cost surface modification strategy to endow UCNPs with good water‐solubility and bio‐compatibility without changing the upconversion efficiency. The intrinsic structure of TWEEN can significantly enhance the water stability of UCNPs via hydrophobic interactions; consequently improving solubility in biological buffers and cell culture media. DOX physically adsorbed on the hydrophobic surface of TWEEN coated UCNPs through hydrophobic interaction with an aliphatic chain within the structure of TWEEN.^104^ Using the same strategy, hydrophobic OA‐capped NaGdF_4_:Yb/Er@NaGdF_4_ UCNPs were coated by carboxyl groups incorporated Tween‐20 to render them water soluble based on the interactions between the fatty‐acid tails of Tween20‐COOH and the long alkyl chain of OA on the surface of UCNPs. In addition, a mixture of different lipophilic chemotherapy drugs (camptothecin (CPT) and DOX) were efficiently loaded into the water‐soluble UCNPs via hydrophobic interactions. In their design, FA was conjugated onto tween‐UCNPs through amidation between –COOH groups and the –NH_2_ groups in the FA molecules, which facilitated targeted dual‐drug delivery and tri‐modal imaging.^105^ As a result, these multifunctional platforms were employed for in vitro and in vivo concurrent therapeutic application and imaging diagnosis.

#### 3.2.3 Upconverted in situ Photopolymerization to Construct Polymer‐Coated UCNPs

**Figure 4:**
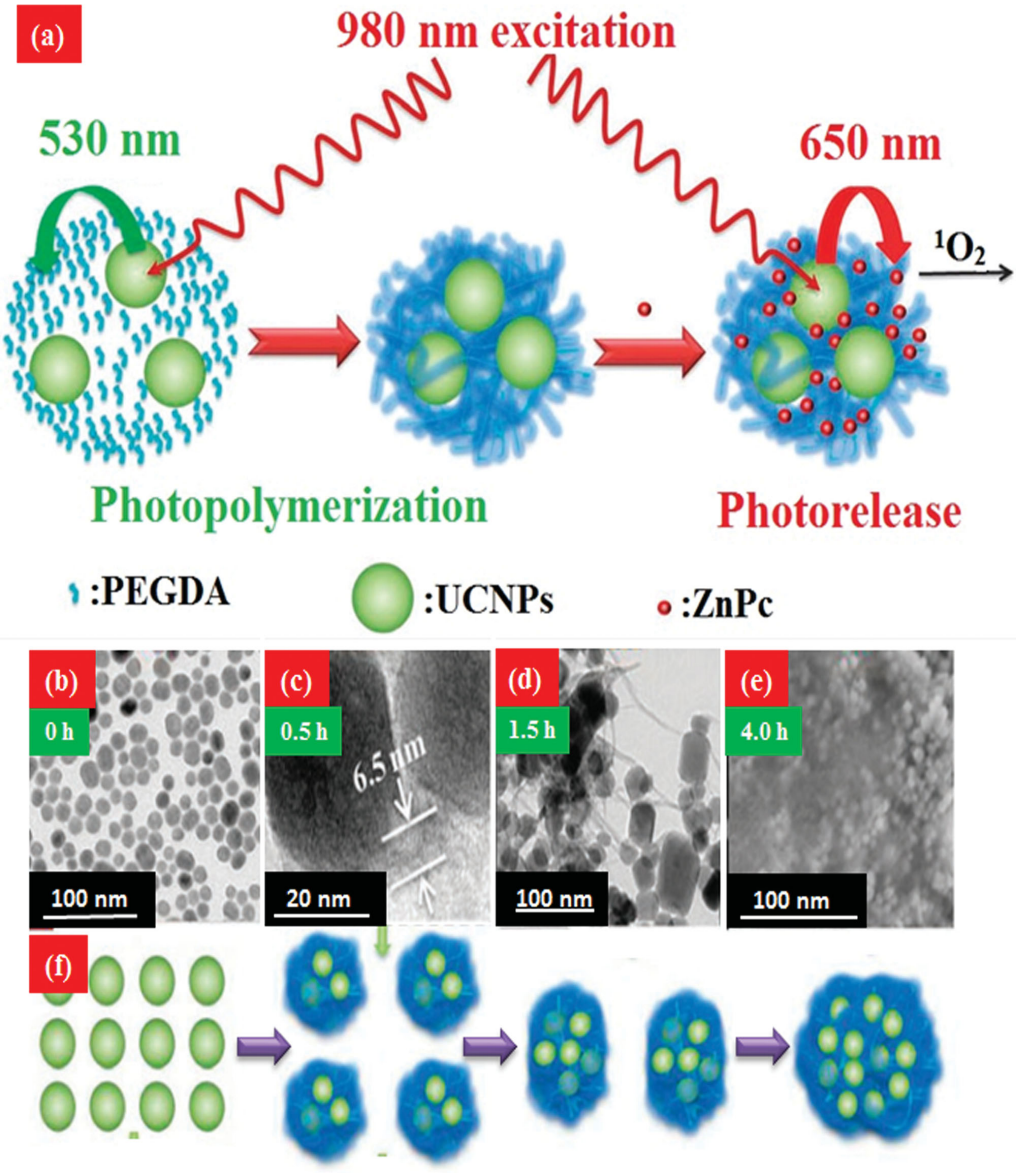
a) Schematic illustration of NIR‐light‐activated photopolymerization of PEGDA hydrogels and photo release of ^1^O_2_ from ZnPc loaded UCNPs–PEGDA hybrid microspheres using multicolor UCL of UCNPs. b–e) TEM and scanning electron microscopy (SEM) images of UCNPs–PEGDA nanocomposites synthesized under 980 nm excitation for 0–4 h; f) Schematic representation of nanocomposites at different photopolymerization stages. Reproduced with permission.^108^ Copyright 2013, Royal Society of Chemistry.

In recent years, a few groups have investigated in situ encapsulation of UCNPs with a hydrophilic polymeric shell by using the upconverted emitted light to initiate a polymerization.^106^, ^107^ Using this technique, Xiao et al. have reported the in situ photopolymerization of the PEG diacrylate hydrogels which was activated with the assistance of NIR‐excited UCNPs as the internal light source. Based on their design, NIR‐light initiated photopolymerization and drug delivery were concurrently achieved. The OA‐capped NaYF_4_: Er,Yb UCNPs were then surface functionalized with hydrophilic ligands of PAA in order to impart water solubility. The upconverted green emission (between 520 and 554 nm) initiated the photopolymerization of PEGDA hydrogels by employing comonomer N‐vinylpyrrolidone (NVP) and Eosin Y as a photoinitiator, which has a strong absorption band between 460 and 540 nm. During this polymerization, photosensitizers were encapsulated. As the red emission band from UCNPs overlaps with the absorption spectra of zinc(II) phthalocyanine (ZnPc) (650–680 nm), the UCNPs‐based system was also utilized for photo‐activated controlled drug delivery of the photosensitizer ZnPc to release singlet oxygen (O_2_) for photodynamic therapy (PDT) application. Besides, in situ polymerized hydrogels can firmly adhere to tissue structures at the injury sites, which make them appropriate tissue scaffolds for photochemical tissue bonding (PTB) employed in surgery. Using NIR light can reach deeper tissue with minimal detrimental heat damages, which is an important step forward towards solving limitations associated with UV or visible light required as excitation source for PTB or PDT (**Figure** **4**).^108^

Following the same concept, in 2014, Haupt et al. employed OA‐capped UCNPs as an internal light source for photopolymerization of a thin polymer shell in situ around the nanoparticles and subsequently endowed UCNPs with various surface properties based on the nature of the utilized monomer. Various water‐soluble polymer shells were synthesized by employing 2‐hydroxyethyl methacrylate (HEMA) and the cross‐linker N,N′‐ethylenebis‐ (acrylamide) (EbAM) hydrophilic monomers with the potential of further bioconjugation through addition of functional monomers (such as glycidyl methacrylate and propargyl acrylamide) to the prepolymerization mixture.^106^ For polymerization, a visible‐light emitter (NaYF_4_:Yb^3+^/Er^3+^) and a UV light emitter (NaKYF_4_:Yb^3+^/Tm^3+^) UCNPs were employed to activate the UV (benzophenone/triethylamine) and visible (eosin Y/triethylamine) initiators, respectively. Using the same in situ photopolymerization can provide a second polymeric shell.^106^ Therefore, this technique has the potential for preparation of water‐soluble functionalized UCNPs by in situ photopolymerization for applications in biomedical and particularly drug delivery.

#### 3.2.4 One‐Step Synthesis of Hydrophilic UCNPs by Polymer Encapsulation

In the one‐step technique, polymers are directly coated on the surface of UCNPs to render them water‐soluble. Thus far, various types of polymers such as PEG,^109^ PAA,^110^ and PEI^111^ have been employed in one‐pot synthetic processes. In 2006, Wang's group reported the first demonstration of one‐step synthesis of water‐dispersible and biocompatible PEI encapsulating NaYF_4_:Yb^3+^ Er^3+^/Tm^3+^ UCNPs. PEI is a highly branched hydrophilic polymer surfactant, with primary, secondary and tertiary amino groups and an overall positive charge. The positively charged amino groups stabilize the UCNPs in solution and can covalently bond to biomolecules. In addition, this organic polymer showed considerable thermal stability, which makes it an excellent candidate for one‐step synthesis reactions at relatively high temperatures.^111^ Following this viewpoint, Hao's group developed a one‐pot synthesis technique to produce water soluble UCNPs with surface functional groups. In their design, 3‐mercaptopropionic acid, 6‐aminocaproic acid and PEG were used to functionalize the surface of nanoparticles by –COOH, –NH_2_, –SH or –OH groups. Functionalized nanoparticles showed strong upconverted emissions in aqueous phase with no discernible cytotoxicity.^109^

## 4 NIR Light‐Activated Release of Drugs from UCNPs‐based DDSs

Therapeutic applications of UCNPs have witnessed rapid growth for drug delivery applications in the past few years. Various types of UCNPs‐assisted delivery systems have been exploited as drug carriers comprising of (i) luminescence‐monitored DDS, (ii) pH‐responsive DDS, (iii) thermo‐responsive DDS, (iv) redox‐responsive DDS, (v) NIR light‐initiated DDS and (vi) targeted DDS.^28^, ^29^, ^30^, ^31^ It is worth mentioning that using UCNPs‐based nanoplatforms as drug carriers facilitates tracking and monitoring of the quantitative drug release in real time by the alteration in the intensity of the anti‐Stokes emissions. A significant body of literature exists in this field already.^112^, ^113^, ^114^, ^115^, ^116^, ^117^, ^118^ For instance, Lin's group used the correlation between upconverted luminescence intensity of NaYF_4_:Yb/Tm and the extent of DOX release as an optical probe to confirm the DOX conjunction and monitor the drug release.^112^ In addition, Lin and co‐workers monitored the release amount of ibuprofen (IBU) from different UCNPs‐based carrier systems based on the quenching of the upconverted luminescence due to the existence of organic groups in IBU with high quenching effect.^117^, ^118^

In particular, the NIR‐initiated drug delivery concept is generating high interest as a prospective candidate to revolutionize light‐activated carriers and overcome the problems associated with conventional light‐activated DDSs.^32^ In general, to fabricate an efficient UCNPs‐based NIR‐trigger controlled drug release system, three essential properties should be taken into account for the structure design; firstly, UCNPs with strong UV/visible upconverted emission, secondly, integration of suitable photoresponsive material and finally, NIR excitation. Remarkable advantages of UCNPs‐assisted light‐activated carriers make them preferable over other conventional light‐activated delivery systems including (i) enhanced biological tissue penetration depths realized by NIR excitation, (ii) less photodamage to live organism achieved by lower excitation light energy, (iii) non‐photodamaging to DNA/RNA; and (iv) good biocompatibility and no evidence of biotoxicity demonstrated by set of in vitro and in vivo toxicology investigation.^36^, ^37^ Furthermore, UCNPs demonstrated non‐blinking activities and showed great stability against photochemical degradation.^42^, ^119^ Various types of pharmaceutical cargo (such as small anticancer chemotherapeutic, biomacromolecules, gene products, nontherapeutic agents, and cells) have been incorporated into the NIR‐initiated DDSs with either covalent or non‐covalent bonds. NIR‐light initiated DDSs are broadly classified into two main categories depending on the nature of the photoresponsive moieties:(i) NIR light‐induced photo releasing of photoactivatable or ‘caged’ drugs and (ii) NIR light‐induced photoswitching of molecules between two structurally and electronically distinct isomers.

### 4.1 NIR Light‐Activated Photolysis of Photocleavable or ‘‘Caged’’ Molecules

UCNPs‐assisted light‐initiated payload releasing systems through photocleavage are designed based on the integration of photocleavable protecting groups (PPGs) within the structure of the drug carriers. PPGs utilized in this kind of system are cleavable upon UV/visible light irradiation and this special photosensitive feature provides spatially and temporally controlled “on‐command” drug delivery.^11^, ^12^, ^120^

In recent years, NIR‐to‐UV/visible UCNPs have been successfully used for activation of various photocaged molecules like D‐luciferin,^16^ carboxylic acid,^11^ nitric oxide (NO),^121^, ^122^ biomolecules,^65^ small interfering RNA (siRNA),^63^ 5‐fluorouracil (5‐FU)^123^ and cell adhesion molecules.^124^ For example, Carling et al. demonstrated NIR‐initiated cage release of carboxylic acid via decoration of UCNPs with photocleavable compounds. In this work, NaYF_4_:TmYb@NaYF_4_ nanoparticles were loaded with 3′,5′‐di(carboxymethoxy)benzoin acetate while its absorption partially overlaps (*λ* = 282 nm) with the anti‐Stokes emission of UCNPs (*λ* = 290 nm). Upon exposure of UCNPs to a 980 nm continuous wave (CW) photon source (556 W cm^–2^), carboxylic acid released in a controlled manner through the photolysis of the cage compound.^11^

**Figure 5:**
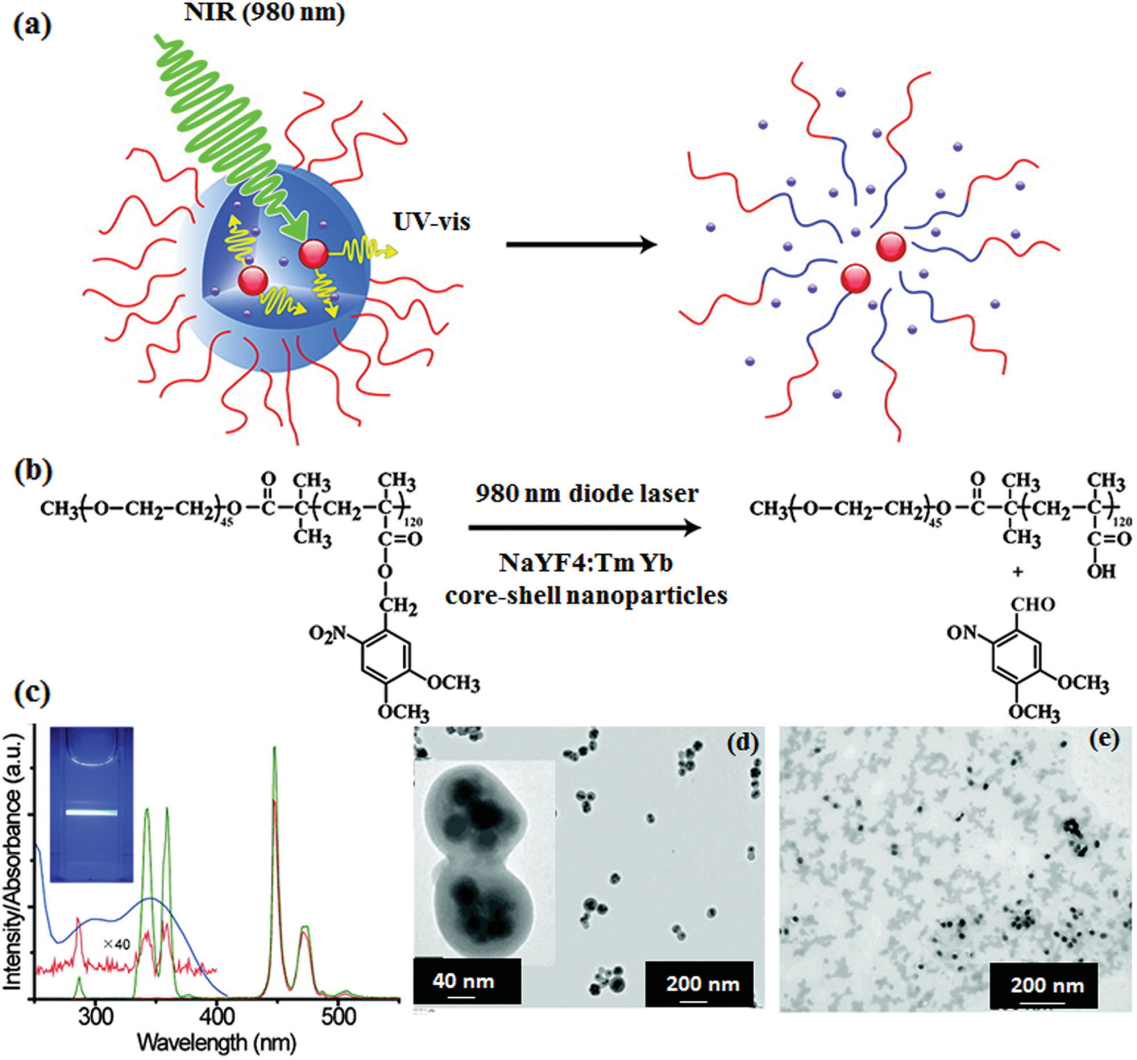
a) Schematic illustration: using NIR light excitation of UCNPs to trigger dissociation of BCP micelles. b) NIR light‐triggered photoreaction with the used BCP of PEO‐b‐PNBMA and UCNPs of NaYF4: Tm/Yb. c) Photographs of UCNP‐loaded micellar solution upon 980 nm diode laser exposure (5 W), its absorption spectrum (blue line), and emission spectra of the neat UCNPs (green line) and the micellar solution (red line). (Emissions of the micellar solution in the UV region being amplified) d) TEM image of UCNP‐loaded BCP micelles before NIR light irradiation, the inset showing two magnified micelles containing several nanoparticles. e) TEM image of the same micellar solution after NIR irradiation (5 W, 4 h), showing the disintegration of BCP micelles. Reproduced with permission.^125^ Copyright 2011, American Chemical Society.

Furthermore, Ford group reported that NIR‐to‐visible NaYF_4_:Yb/Er@NaYF_4_@SiO_2_ upconverting nanocrystals which were coated by poly(allylamine hydrochloride) (PAH) can trigger photoreactions of the NO under 980 nm light illumination, utilizing an iron nitrosyl complex (iron/sulfur/nitrosyl cluster Roussin's black salt anion Fe_4_S_3_(NO)_7_^−^) as the NO precursor.^121^ In addition to the findings discussed above, Zhao and co‐workers demonstrated the feasibility of UCNPs to act as an internal UV source to dissociate the photosensitive block copolymer (BCP) micelles and initiate the release of Nile Red as a hydrophobic model payload. UV‐sensitive BCP comprising of hydrophilic poly (ethylene oxide) (PEO) and a hydrophobic polymethacrylate bearing photocleavable *o*‐nitrobenzyl (PNBMA) groups which its absorption overlaps with the 350 nm anti‐Stokes emission from UCNPs under 980 nm illumination (5 W cm^–2^, 4 h) (**Figure** **5**).^125^

In 2012 another noticeable example was introduced by the same group which was the first demonstration of photoactivation of light‐degradable hydrogel loaded with UCNPs and biomacromolecules (protein and enzyme) under CW NIR light irradiation. The photosensitive hydrogel used in this study was designed with a PEG cross‐linker held together by photosensitive *o*‐nitrobenzyl groups. Under exposure of the designed hybrid UCNP−hydrogel system to the NIR laser (3–5 W), the upconverted emission band (between 250 and 400 nm) induced a photocleavage reaction of the *o*‐nitrobenzyl groups, which resulted in the structural changes in photosensitive hydrogels (breakdown of the entire gel), releasing the entrapped inactive biomacromolecules on the on–off pattern, where their bioactivity could be restored.^65^

Concurrently, tremendous effort has been dedicated to the design and fabrication of UCNP‐mediated DDSs for light‐initiated drug release and bioimaging in vitro and in vivo studies.^16^, ^69^, ^126^ In this context, Xing and co‐workers demonstrated a design for the photolysis of caged d‐luciferin via utilizing bioconjugated UCNPs in vitro and in vivo and performing bioluminescence imaging studies by combining versatile photocaged compounds with the Tm/Yb co‐doped NaYF_4_ core‐shell nanoparticles. In their design, UCNPs were coated with thiolated silane molecules and subsequently conjugated to d‐luciferin that was photocaged with a 1‐(2‐nitrophenyl) ethyl photoresponsive group. The photocaged d‐luciferin molecules were modified with a PEG linker that contained a terminal maleimide group, which is reactive with thiols, in order to provide high efficient loading density of d‐luciferin on the surface of a nanoparticle. Upon excitation with 980 nm NIR, the caged D‐luciferin molecules, which were efficiently blocked by (2‐nitrophenyl)ethyl groups, released from the surface of nanoparticles via the photodeprotection of the 1‐(2‐nitrophenyl)ethyl group owing to the absorption of anti‐Stokes‐shifted UV emission from UCNPs.^16^

Another study, which was carried out at the cellular level, also proved the rational design of remote‐control photo‐release of imaging probes and caged compounds using NIR light and upconverting nanoparticles. Based on their design, NaYF_4_:Yb,Tm@NaYF_4_ UCNPs were first coated with silica shell then, photocleavable *o*‐nitrobenzyl as the linker capped the pore mouths of mesoporous silica; and subsequently, the antitumor drug DOX was adsorbed onto the surface of the prepared mesoporous silica‐UCNPs complex. As the absorption band of the photocaged DOX overlaps with the upconverted emission band of the UCNPs, upon NIR excitation at 980 nm, disassociation of photocaged DOX molecules from the surface of the nanocarriers was triggered, which showed high efficiency of the controlled release of DOX in cancer cells. In addition, the surface of the nanocarriers was functionalized with FA units to enhance the selective targeted and photo‐controlled drug delivery in the tumor cell lines.^69^

**Figure 6:**
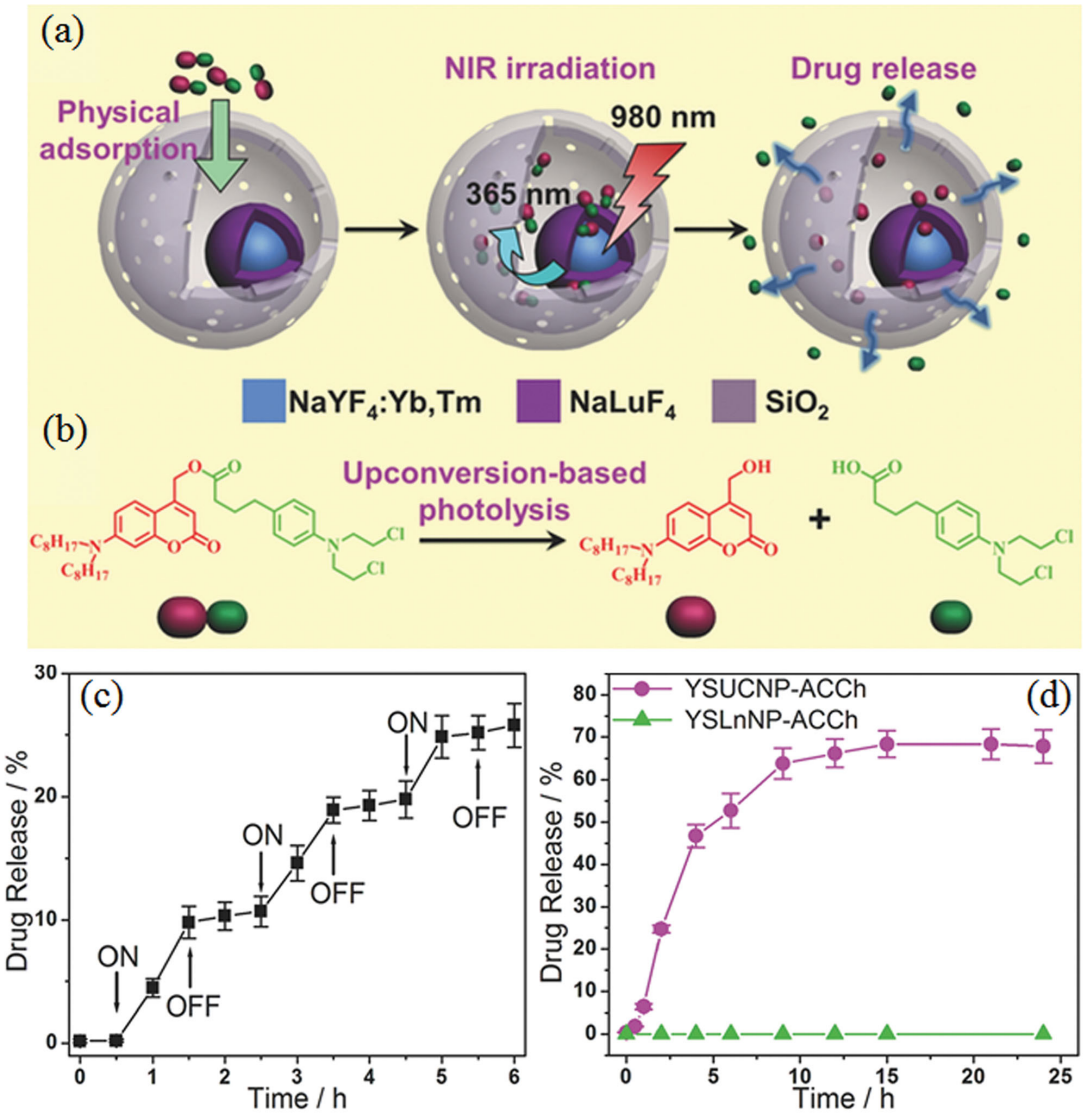
a,b) Schematic illustration of the NIR‐regulated upconversion‐based PDD and the photolysis of the prodrug under upconversion emission from the YSUCNPs. c) The photo‐regulated release of chlorambucil (drug) from YSUCNP‐ACCh controlled by a 980 nm laser. “ON” and “OFF” indicate the initiation and termination of laser irradiation. (980 nm laser at 570 mW cm^−2^). (d) The release profiles of chlorambucil from YSUCNP‐ACCh and YSLnNP‐ACCh (similar yolk–shell nanoparticle consisting of the NaYF_4_:Yb@NaLuF_4_ yolk without the Tm^3+^ activator) in PBS solution (pH = 7.5) under CW 980 nm irradiation. Reproduced with permission.^62^

Li group reported the first successful example of phototrigger‐controlled drug‐release beyond the cellular level, in living animal tumor tissues. In their study, a creative yolk‐shell structured UCNPs (YSUCNPs) with a heterogeneous core–shell lanthanide nanocrystal (NaYF_4_:Tm^3+^, Yb^3+^@NaLuF_4_) as the yolks and mSiO_2_ as the hollow shells was designed and fabricated. The carrier system was loaded with hydrophobic amino‐coumarin as phototrigger and chlorambucil as photocaged antitumor drug. The hollow cavities of this unique structure facilitated substantial loading capacity for anticancer drug molecules and their sustainable release manner. Additionally, amino‐coumarin was modified by two octyl groups to avoid premature release by preventing the diffusion of prodrug from the cavity of the YSUCNPs under physiological conditions. Under excitation at CW 980 nm NIR photon source, the intense anti‐Stokes‐shifted UV luminescence initiated the cleavage of the amino‐coumarin (with maximum absorption at 380 nm), which led to the uncaging and releasing of the drug through pores in the silica shell based on the on–off pattern. The results revealed that their unique YSUCNPs‐ACCh nanoplatforms efficiently liberate the anti‐tumor drug into cancer cells in response to NIR radiation, and thus enhance inhibitory drug action to suppress tumors growth rate and extend the survival period of mice (**Figure** **6**).^62^

In addition to the results discussed above, Chien et al. reported innovative UCNPs‐based light‐controllable targeting nanoplatform in which the PEGylated UCNPs@SiO_2_ were functionalized by FA through interaction of amino groups of FA and carboxylate groups of PEG. In addition, 2‐nitrobenzylamine hydrochloride (NBA) used as cage molecules to mask carboxylate groups of FA through formation of covalent amide bonds with the –NH_2_ groups of the opposite terminus of FA. In this study, DOX was thiolated to form a cleavable disulfide bond on the surface of PEGylated UCNPs@SiO_2_, released through cleavage by lysosomal enzymes within the tumour cells. In this design, 70% of FA was blocked by NBA and liberated by 980 nm light illumination to allow the folate‐PEGylated UCNPs@SiO_2_‐DOX to target cancer cells with improvement in cellular uptake and thus reduction of toxic adverse effects.^64^

**Figure 7:**
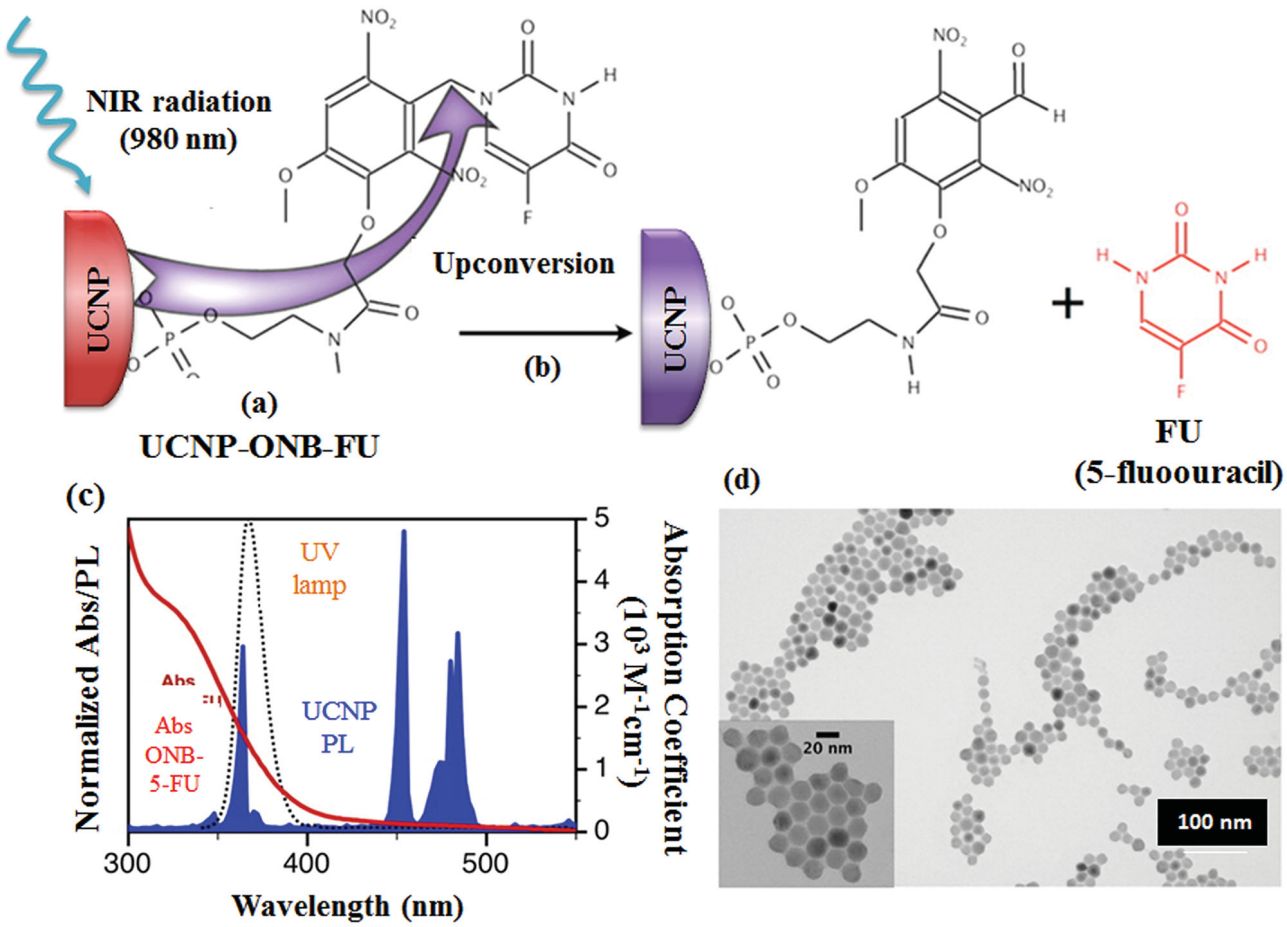
a) Modification of water‐soluble β‐NaYF_4_: 4.95% Yb, 0.08% Tm/β‐NaYF_4_‐doped core−shell o‐phosphorylethanolamine‐capped UCNPs with ONB‐FU. b) NIR excitation (980 nm) of the UCNPs resulted in upconverted PL emission at 364 nm used for photocleavage of the ONB−FU bond and subsequent release of 5‐fluorouracil from the UCNP surface. Drawing is not to scale. c) Normalized PL spectrums of NaYF_4_ (4.95% Yb, 0.08% Tm) core−shell UCNPs under irradiation with 980 nm laser (blue line) and UV−vis absorption spectra of the ONB−FU prodrug (red line). The molar absorption coefficient of the ONB−FU prodrug is 1560 M^−1^ cm^−1^ at 364 nm. The UCNP PL spectrum is normalized to the most intense UV−vis emission at 454 nm. The normalized emission profile of the UV lamp with a maximum at 367 nm used for direct UV photocleavage is shown as a black dashed line. d) TEM image of OA‐capped UCNPs. Reproduced with permission.^123^ Copyright 2014, American Chemical Society.

Controllable liberation of chemotherapeutic 5‐FU from photocleavable UCNP−*o*‐nitrobenzyl−FU conjugate was reported by Krull's group.^123^ In their design, *o*‐phosphorylethanolamine ligand was coated on the surface of UCNPs, which played double roles; on the one hand, it rendered nanoparticles water soluble by using a ligand exchange process, which displaced the hydrophobic OA coating with water soluble ligands. On the other hand, the amine functional group of this ligand provided the conjugation of carboxylic group of *o*‐nitrobenzyl‐FU prodrug on the surface of UCNPs capped *o*‐phosphorylethanolamine via carbodiimide chemistry. Moreover, the compactness of this ligand as compared with other coating units such as PEG and silica shell facilitated close enough proximity between photocleavable prodrug and UCNPs (<2 nm). This minimal distance between emitter and photolabile group is beneficial for efficient energy transfer and thus using UCNPs as internal trigger to release the 5‐FU (**Figure** **7**).^123^

### 4.2 NIR Light‐Activated Photoswitching of Molecules between Two Isomers

A breakthrough has been achieved by another approach for the architect design of the UCNPs‐based light‐initiated DDSs in vitro and in vivo is using the photochromism phenomenon via integration of photoswitchable molecules into the structure of the carrier system.^127^ Owing to the weaknesses of using direct UV or visible light to induce the photoreaction, NIR has emerged as an appealing alternative to trigger photochemical isomerization of photochromic through using UCNPs. It should be mentioned that photochromic compounds used in UCNPs‐mediated photoresponsive carriers should have overlapped absorption band with UV or visible emission generated from UCNPs. From the viewpoint of materials and structure, hexagonal NaYF_4_ nanocrystals doped with lanthanide ions, are one of the most ideal tools for “remote control” photoswitching and under absorbing NIR light depending on their constituents, they can emit ultraviolet, violet, blue, green, and red light.^128^, ^129^, ^130^

Following this concept, two kinds of UCNPs, which emit UV (NaYF_4_:TmYb) and visible light (NaYF_4_:ErYb) under NIR exposure, were employed to trigger the photochemical switch of dithienylethene (DTE) molecules between their ring opening and ring closure reactions. The ring‐open isomer of DTE absorbs UV light (365 nm) produced by UV‐emitting NaYF_4_: Tm Yb nanoparticles under NIR light excitation and undergoes conversion to its ring‐closed isomer while the reverse reaction (ring‐opening reaction) is initiated by visible light (>450 nm) generated by the green‐emittance of the NaYF_4_:ErYb under NIR light. Thus, both of the UCNPs and DTE should remain in close enough vicinity to promote reabsorption of the upconverted emission band from the nanocrystals by the photochromic molecules. For this reason, components were casted in a polymer composite material consisting of cross‐linked poly (ethyleneglycol) dimethacrylate and toluene, which provides a flexible environment for the “remote control” photoswitching.^129^

In a complementary study carried out by Branda group, it was reported that the “remote control” photoswitching using NIR light is as equally effective as the direct photoreaction of the DTE derivatives with UV and visible light, although the reaction rates are slower.^131^ It must, however, be noted that these successful findings as a proof‐of‐concept opened a door towards development of “remote control” photoswitchable drug delivery carriers using UCNPs‐containing structure. As demonstrated in the literature, knowing the reversible trans–cis photoisomerization property of azobenzene, Liu and co‐workers rationally architected NaYF_4_: Tm/Yb UCNPs coated with azobenzene‐containing mesoporous silica, which was loaded with chemotherapeutic drug DOX as the very first NIR‐trigger‐controlled drug‐release for cancer therapy from a UCNPs‐based DDS.^2^, ^127^ In this system, DOX was loaded into the system by forming hydrogen bands with the surface silanol groups of silica to prevent premature release in aqueous environments while TAT peptide was conjugated on the outer surface of nanoparticles to enhance the cellular uptake. UV light irradiation of an azobenzene molecule triggers the transformation of the planar *Trans* isomer to the bent *cis* isomeric form while the reversible reaction takes place under irradiation of visible light. As the upconverted emission wavelength of the nanocrystals (*λ* = 350 nm, *λ*_2_ = 450 nm) partially overlaps with the absorption wavelength of the azobenzene molecules (*λ*_1_ = 330 nm,*λ*_2_ = 440 nm), excitation of the UCNPs with NIR laser light can cause the photoisomerization of the azo molecules. The back and forth wagging motion of the azobenzene molecules acted as a molecular impeller that resulted in releasing of DOX. In addition, concurrent emitted upconverted NIR light endows potential in vivo luminescent imaging of whole‐body animals. It is worthwhile to note that in this study, the amount of the released anticancer molecules was well‐tuned by altering the time duration and intensity of NIR light exposure, which demonstrated NIR light‐regulated precise drug release.^86^

## 5 Incorporation of Targeting Functionality into UCNPs‐based DDSs

To date, major breakthroughs have been successfully accomplished in the area of UCNPs‐based DDSs for remote‐controlled release of therapeutic payloads. Another challenge for enhancing therapeutic effects is to direct the bioactive compounds to the target site in order to minimize the adverse side effects on healthy cells/tissues that may arise from systemic distribution of bioactive compounds throughout the blood stream.^132^, ^133^, ^134^ Non‐specific bio‐distribution can cause cytotoxicity to healthy and cancer cells alike, causing severe toxic side effects in attempts to achieve sufficient anti‐cancer efficacy. Therefore, various UCNP‐based nanocomposites have been exploited as targeted DDSs to diminish toxic adverse effects and improve remedial efficacy. Currently there are two principal schemes of drug targeting for UCNPs‐based DDSs. The first involves the use of magnetic compounds and the action of an external magnetic field to direct the bioactive compound to the desired site. In the second approach, targeting is achieved using a targeting moiety with high affinity toward the site of interest.^100^, ^133^, ^135^ It should be mentioned that due to the ability to condense light from a laser source into a highly focused beam, UCNPs by themselves also have the potential to be used as targeted delivery tools in vivo.^123^

### 5.1 UCNPs‐based DDSs with Targeting Moieties

Targeting using specific ligands or biomolecules possessing target‐specific recognition is the most frequently used approach to fabricate targeted carrier systems.^136^ These acceptor‐labeled molecules, such as antibodies, lectins, charged molecules, FA and TAT peptide, can specifically recognize the receptor on the surface of target cells to endow targeting capability to DDSs. Targeting agents can be either directly incorporated into the UCNPs‐based DDSs or via the use of polymeric materials.^100^, ^137^, ^138^ To date, most of the targeting research is related to the use of UCNPs for imaging rather than drug delivery, with a few exceptions.

Lin's group biologically functionalized core and shell structured *β*‐NaYF_4_:Yb^3+^, Er^3+^@mSiO_2_‐PEG nanoparticles through grafting FA onto the surface of silica by covalent linking. FA imparts specificity and affinity toward folate receptors on the surface of cancer cells in order to direct DOX to the cancer cells via a receptor‐mediated endocytosis process. The results revealed that the cellular uptake of DOX by Hela cells is almost two times as high as non‐modified nanoparticles, which demonstrated the targeted proficiency of designed UCNPs‐based nanocarriers.^139^ A follow‐on study, where FA is directly grafted onto the surface of hollow, mesoporous structured α‐NaYF_4_:Yb^3+^, Er^3+^ UCNPs through amide reaction produced similar results.^97^ Interestingly, in both studies, the green emission produced by the UCNPs enabled the cell endocytosis process to be monitored. Following this approach, Chien et al. tagged NaYF_4_: Tm/Yb UCNPs with DOX and FA to design a nanoplatform for light‐controllable on‐demand targeting chemotherapy and bioimaging. In this approach, targeting agent, FA, was masked with NBA. FA groups were liberated by 980 nm light illumination which allowed the folate‐PEGylated UCNPs@SiO_2_‐DOX to target cancer cells which proved the potential in the enhancement of selective targeting and hence minimizing toxic side effects of chemotherapy.^64^

UCNPs has also been coated with antigen to enable their use in immunotherapy treatment.^100^, ^140^ Xiang et al. engineered an advanced dendritic cell (DC) vaccine utilizing ovalbumin (OVA)‐coated UCNPs for initiating CD8^+^T cell immunity for immunotherapy. In this design, dual‐polymer‐coated UCNPs dramatically enhanced the uptake of OVA in DCs and eventually induced DC maturation and cytokine release. The UCNPs showed potential for monitoring the in vivo translocation of DCs which was the first example of highly sensitive in vivo DC tracking.^100^

### 5.2 Magnetic Targeting of UCNPs‐based DDSs

Besides target molecular recognition, using magnetic targeting has seen a rapid evolution in the development novel DDSs. The principle behind using magnetic materials in UCNPs‐based DDSs is to direct the drug‐loaded UCNPs to specific organ or tissue in the body by applying extracorporeal magnets. Gai et al. fabricated a magnetic core; UCNPs shell nanocomposites via a sol–gel process. The magnetite (Fe_3_O_4_) core was encapsulated by a non‐porous SiO_2_ layer in order to prevent fluorescence quenching by the magnetite. A second mSiO_2_ layer was utilized to accommodate IBU as the drug model. Finally, a NaYF_4_:Tm/Yb UCNPs layer was deposited on the outer layer of the mSiO_2_. Targeted drug delivery under an external magnetic field (38.0 emu g^–1^), controlled release of the drug, and bright UC emission under NIR light exposure were shown to be feasible for the developed targeted UCNPs‐based DDSs.^77^

## 6 Effects of NIR Light Power Intensity on the Upconverting Phenomena and Activation of Drug Release from Yb^3+^‐sensitized UCNPs

Most of the reported UCNPs to date have been doped with Yb^3+^ ions as the sensitizer, which has the maximum absorption band at around 980 nm.^141^ Therefore, NIR light at 980 nm is used for the initiation of therapeutic payload release in UCNPs‐based DDSs. The laser power intensity and illumination time period influence the upconverted emission spectra, which in turn affects the quantitative drug release behavior.^36^, ^119^, ^142^ In 2007, Capobianco's group reported the non‐linear power dependencies of Tm^3+^ and Er^3+^ anti‐Stokes emissions at low power densities. At high (but unspecified) excitation densities, the upconversion power dependency became linear due to saturation of the upconversion processes.^143^ Yan's group later demonstrated the saturation effects of the Yb^3+^ and Tm^3+^ photon absorption.^52^ Zhang's group reported the increase in upconverted fluorescence signal as a result of power intensification. In their study, core and shell (NaYF_4_:Yb,Er/Tm@SiO_2_) nanospheres showed observable upconverted fluorescence under excitation of nanoparticles with minimum applied power density of 1 W cm^–2^ up to maximum of 50 W cm^–2^ (saturation limit).^144^

In follow‐on studies, several groups have investigated the effects of laser power density and irradiation time on the quantitative drug release from NIR‐initiated DDSs. In 2012, Zhang et al. demonstrated that the quantitative release of caged NO form NaYF_4_:Yb/Er@NaYF_4_ nanoparticles is a linear function of the 980 nm laser illumination period while operating at constant power of 1W (with a slope of 2.4 pmoles s; interval 2 to 160 s).^121^ However, upconverted emissions and dosage of the released NO were reported to be non‐linear in response to systematically variation in the output power of the excitation light from 1 to 4.5 W.^121^ Likewise, Zhang et al. demonstrated an analogous trend of the NO release from different drug delivery vehicles (UCNPs@SiO_2_@m‐SiO_2_) with linear response to the irradiation time as opposed to a nonlinear response to variations in the NIR power intensity.^121^ Based on their findings, log (NO) vs log (laser power) plot presented a slope of 1.44.^122^

Shi's group also demonstrated the nonlinearity response of drug release to power density changes. The maximum amount of DOX (80 wt%) was released from NaYF_4_:TmYb@NaYF_4_@mSiO_2_ nanoparticles under exposure to 8.9 W cm^–2^ 980 nm NIR laser for 16 h, while under intermittent NIR exposures with 2.4 W cm^–2^ (identical irradiation time) 40 wt% drug release was achieved.^86^ Furthermore, in the intracellular drug delivery studies, Yeh's group demonstrated that the degree of cell viability is dependent on both the irradiation time and NIR power intensity. Under 980 nm NIR (11 W cm^–2^) irradiation of NaYF_4_:Yb,Tm@SiO_2_‐DOX for 1 min, Hela cells viability was reduced to approximately 57% (at 2.67 μM DOX); the cell viability dropped to approximately 50% in response to 9.6 W cm^–2^ of 980 nm irradiation for 2 min.^64^ In general, the kinetics of the photoreaction is proportional to power of the incident photon source and thus the release of payload can be easily tuned by changing the power density and irradiation period of the NIR photon source.^123^

Although Yb^3+^‐sensitized UCNPs is promising as a light source for photo‐triggered controlled drug release, there are several limitations that need to be overcome before its practical use becomes a reality. Yb^3+^ ions have low absorption at 980 nm (quantum yield <1%), especially under low‐power intensity laser illumination. Most of the photoresponsive moieties that were used in DDSs have absorption bands in the UV region, thus requiring five or four photon NIR‐to‐UV upconverting pathways to initiate the photoreaction and thus therapeutic release.^35^, ^145^, ^146^, ^147^ Thus high power intensities (typically between several mW.cm^−2^ to several hundred W cm^–2^, see **Table** **2**) and prolonged radiation time are required for excitation.^77^, ^148^ For example, Zhao's group used very high power 980 nm photon sources (3–5 W) for irradiation time interval (from 30 min up to 4 h) to provide UV upconverted emissions in order to release different payloads (biomacromolecules and Nile red) from UCNPs‐based DDSs (hydrogel loaded with UCNPs^65^ and block copolymer micelles loaded UCNPs,^125^ respectively). High power intensity can have a deleterious effect on the patient. Water‐rich tissues will transform the light energy into large amounts of thermal energy, which thus hinders the use of the DDSs in vivo.^62^, ^149^ Based on the “American National Standard for Safe Use of Lasers”, the extreme permissible exposure of skin to NIR light is 0.726 W cm^–2^.^150^

**Table 2:** Summary of reported UCNP‐mediated photoreaction

UCNP platform	Light responsive compounds	Photoreaction	The spectral overlap between UCNPs (*λ*_em_) andlight‐responsive moieties (*λ*_abs_)	Intensity[W cm^–2^]
NaYF_4_:Yb/Tm @NaYF_4_	*o*‐nitrobenzyl derivatives	Photo‐cleavage	350	2.8,^177^ 255,^16^ 5.6,^69^ Unknown^125^
NaYF_4_:Yb/Tm	2‐nitrobenzylamine hydrochloride	Photo‐cleavage	343	11 and 9.6^64^
NaYF_4_:Yb/Tm	4‐(hydroxymethyl)‐3‐nitrobenzoic acid	Photo‐cleavage	360	2–6^124^
Na(Y/Gd)F_4_:Yb/Er/Tm	Fe_4_S_3_(NO)_7_^−^	Photolysis	540	100–400^122^
NaYF_4_:Yb/Tm@NaYF_4_	3′,5′‐ di(carboxymethoxy)benzoin acetate	Photolysis	290	556^11^
NaYF_4_:Tm/Yb@NaLuF_4_	Amino‐coumarin	Photo‐cleavage	380	0.05^62^
Various UCNPsa)	Dithienylethene	Isomerization	300–350b)	150–500b), 129, 131
			520–620c)	15c), 131
NaYF_4_:Tm/Yb@NaYF_4_	Azobenzene	Isomerization	330	2.4, 6.3 and 8.9^86^
NaYF_4_:Nd/Yb/Tm@NaYF_4_:Nd@NaYF_4_@	Spiropyran/merocyanine	Isomerization	342d)	40d), 79
NaYF_4_:Yb/Er			560e)	15e), 79
NaYF_4_:Yb/Tm	Azotolane	Isomerization	385	15^178^

^a)^core@shell and core@shell@shell NaYF_4_ UCNPs;

^b)^ring‐open isomer;

^c)^ring‐closed isomer;

^d)^ring‐opening reaction of the spiropyran(SP) to merocyanine (MC) driven by the excitation at 808 nm;

^e)^ring‐closing reaction of the MC to SP driven by the excitation at 980 nm.

To date, few researchers have attempted to control and alleviate the overheating concerns. Qu and co‐workers reported on demand adhesive cell release from UCNPs using short interval exposure (2.5 min break after 2.5 min irradiation) to 2–6 W cm^–2^ 980 nm laser with less than 3 °C increase in water temperature.^124^ Yeh et al. reported that the temperature of the water increase to only 37.7 °C upon exposure of cancer cells to a 11 W cm^–2^ 980 nm laser for 1 min.^64^ Nonetheless, as the quantitative drug release is a function of laser irradiation time, low illumination duration could result in insufficient drug release from the UCNPs‐based carrier systems. In order to bridge the gap between the developed NIR‐initiated DDSs and clinical practice, research has been directed towards (i) improving quantum yield of UCNPs through shell coating; (ii) incorporation of photoresponsive materials into UCNPs that can be activated with upconverted light emitted from UCNPs under ultralow‐intensity 980 nm light source; and (iii) development of new generations of UCNPs with tunable excitation wavelength, which potentially can be excited by a more biocompatible ≈800 nm excitation wavelength (Nd^3+^‐sensitized UCNPs) or a broadened excitation wavelength range (dye‐sensitized UCNPs). The preceding sections will focus on work directed towards tuning the excitation/emissions intensity and wavelength of NIR‐initiated carrier systems.

### 6.1 Enhancement in Quantum Yield of UCNPs through Core and Shell Design

**Figure 8:**
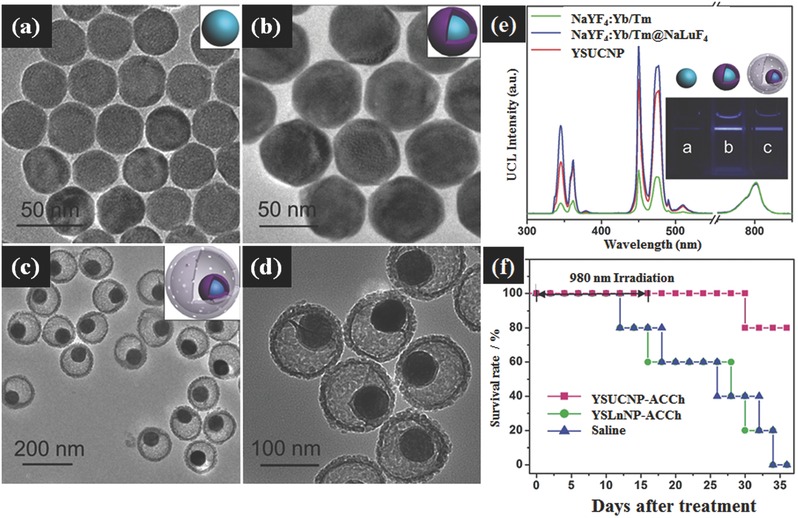
TEM images of the a) NaYF_4_:Yb,Tm, b) NaYF4:Yb,Tm@NaLuF_4_, and c,d) YSUCNP nanoparticles. e) Upconversion emission spectra of the NaYF_4_:Yb,Tm (green line), NaYF_4_:Yb,Tm@NaLuF_4_ (blue line) and YSUCNP (red line) nanoparticles under excitation at 980 nm. The upconversion emission intensities were normalized to the Tm emission at 800 nm (^3^H_4_→^3^H_6_). Inset: upconversion emission photos of the NaYF_4_ :Yb,Tm(a); NaYF_4_:Yb,Tm@NaLuF_4_ (b); and YSUCNP nanoparticles (c) in cyclohexane suspension with equal concentration of NaYF_4_:Yb,Tm (1 mg mL^−1^).f) Survival rate in mice intratumorally injected with 0.04 mL of 10 mg mL^−1^ YSUCNPACCh (purple line), YSLnNP‐ACCh (similar yolk–shell nanoparticle consisting of the NaYF_4_:Yb@NaLuF_4_ yolk without the Tm^3+^ activator/green line) and 0.04 mL saline (blueline) on the 1st day and on the 9th day. In all mice groups, the tumors were exposed to 980 nm laser (50 mW cm^−2^) for 20 min each day for 16 days. Reproduced with permission.^62^

A way to circumvent the need for using a relatively high power photon source is to enhance the quantum yield of UCNP by coating the nanoparticles with either metal fluoride shells (doped or undoped) or shells of different compositions such as CaF_2_. Typically, for nano‐sized UCNPs, a large proportion of the dopant ions are distributed on the surface of particles as a less symmetric crystal field.^26^ Emission spectrum from these nanoparticles can be easily quenched by non‐radiative energy loss and high energy oscillators caused by surface defects, impurities, ligands, and solvents.^9^, ^151^ Furthermore, excited interior ions can transfer their energy to the surface through neighbor ions and consequently disappear non‐radiatively.^152^ Energy loss can be suppressed, and deleterious surface quenching effects can be minimized, by coating the nanoparticles with a uniform shell to enhance the quantum yields (lessen the non‐radiative decays).^26^, ^44^, ^48^, ^153^ In this regard, Chow's group coated hexagonal phase nanoparticles of NaYF_4_:Yb, Er/Tm with an undoped NaYF_4_ shell, which showed upconverting emissions enhancement of 7.4 times from NaYF_4_:Yb,Er@NaYF_4_ and 29.6 times from NaYF_4_:Yb,Tm@NaYF_4_.^47^ Liu's group explored the crystalline shell effect in 10 nm NaGdF_4_:Yb/Tm nanoparticles coated with a 2.5 nm NaGdF_4_ undoped shell which showed a nearly 450‐fold enhancement of the anti‐Stokes emissions upon 980 nm excitation (10 W/cm^2^).^154^ Following this approach, Li's group have successfully initiated caged anticancer drug (chlorambucil) release from UCNPs upon excitation with ultralow operating power intensity (50 mW cm^–2^) 980 nm laser by designing yolk‐shell (yolk:NaYF_4_:Tm/Yb@NaLuF_4_ and shell:mSiO_2_) structured UCNPs. These nanoparticles enhanced the UV range emissions (345 nm) by 4.3 fold compared with the core (NaYF_4_: Tm/Yb), which consequently improved the response of the photocleavable amino‐coumarin (absorption peak at 380 nm) to upconverted emissions (**Figure** **8**).^62^ Upon exposure of these nanoparticles to a 980 nm photon source under a power density of 570 mW cm^–2^, chlorambucil effectively released from the coumarin derivative, resulting in >50% release of the drug within 6 hours, and a maximum release of about 68% of the drug after 15 h.^62^ Likewise, Krull and co‐workers proved that the irradiation of core−shell UCNPs (β‐NaYF_4_:Yb/Tm@β‐NaYF_4_) with low power 980 nm laser (10, 30, and 80 mW and corresponding power densities of 7.9, 23.7, and 63.2 mW cm^–2^, respectively) for different irradiation time (0−80 min) effectively caused photocleavage of *o*‐nitrobenzyl to liberate the chemotherapeutic 5‐FU. In particular, upon absorbing 980 nm light at 23.7 mW cm^–2^ (≈14 min) upconverted UV‐blue emissions initiated complete interfacial release of 5‐FU.^123^

In addition to the rare earth fluoride based shells, heterogeneous core@shell structures (core and shell of different compositions) have attracted special attention for the enhancement of the upconverted emission efficiency. Recently, CaF_2_ shell has emerged as a great candidate to replace the commonly used NaYF_4_ shell due to its optical transparency, biocompatibility, stability, high crystallizability and negligible lattice mismatch with NaYF_4_.^155^, ^156^, ^157^, ^158^, ^159^ For example, Wang et al. demonstrated a facile way to fabricate NaYF_4_:Ln^3+^@CaF_2_ nanocrystals with ≈300‐fold enhancement of upconverted emission yield compared with the parent core NaYF_4:_Ln^3+^. Particularly, a shell of CaF_2_ enhanced anti‐Stokes emissions from NaYF_4_:Yb,Er (10–13 nm) by a factor of 4 to 5.^155^ Another striking example was reported by Han's group in which NaYbF_4_:Tm@CaF_2_ nanoparticles highly enhanced NIR_980_‐to‐NIR_800_ upconverted photoluminescence 35 times stronger than that of NaYbF_4_:Tm, elevating quantum yield to be as high as 0.6% under low energy excitation of 0.3 W cm^–2^.^160^ Han and co‐workers extended usage of such heterogeneous core@shell nanoparticles (α‐NaYF_4_: (20–99.5%) Yb, Tm@CaF_2_) to realize tunable enhancement of NIR‐to‐UV anti‐Stokes emissions by two orders of magnitude compared with the core nanoparticles. The CaF_2_ shell showed greater resistance to quenching problems induced by aqueous medium compared with the widely used NaYF_4_ shell which resulted in preserving upconverted UV emissions.^161^ Furthermore, Han's group developed a high Yb^3+^ doped NaYF_4_: Yb (80%), Er(2%)@CaF_2_ nanoparticles, leading to a 15‐fold enhancement of NIR‐to‐red light compared with their β‐phase counterparts, reaching a quantum yield as high as 3.2%. These optimal nanoplatforms with amplified upconverted red emission were demonstrated as promising carriers for photodynamic therapy under a biocompatible excitation power density of 0.5 W cm^–2^.^157^ These results demonstrated that through enhancing the quantum yield of core–shell structured UCNPs, low range of 980 nm intensity can be sufficient to generate required upconverted emissions for exciting the nanoparticles and eventually initiate the photoreaction. These approaches can avoid undesired excessive heat effects generated by high intensity laser illumination and can open new possibilities for more biocompatible delivery systems that can be employed in clinical and medical setups.

It is noteworthy to mention that the energy migration‐mediated upconversion processes were also investigated to enhance the upconversion efficiency by using a host lattice with low phonon energy (such as gadolinium (Gd) sublattice). Through the Gd^3+^ sublattice, the excitation energy can migrate with fast rates over a long distance in favor of energy transfer processes.^152^, ^162^ Energy migration‐mediated upconversion in core/shell nanoparticles synergistically combines the advantages of a core/shell structure and the fast energy migration effect, enhancing the anti‐Stokes emissions.^163^, ^164^ In a demonstration by Liu and co‐workers, efficient upconversion emission was realized through rational design of a core@shell structure with a set of lanthanide ions incorporated into separated layers at precisely defined concentrations, through using Gd sublattice‐mediated energy migration for a wide range of lanthanide activators (Eu^3+^, Tb^3+^, Dy^3+^, and Sm^3+^) without long‐lived intermediary energy states.^162^ In this design, the core/shell structure eliminates deleterious cross‐relaxation while the excitation energy can efficiently migrate over the Gd sublattice for a substantial distance to the activators, which results in an enhanced upconversion efficiency. In another study carried out by Liu's group, the surface quenching of the migrating energy in NaGdF_4_:Yb/Tm@NaGdF_4_ nanoparticles was suppressed by using an inert NaYF_4_ layers which enhanced the energy trapping by the activators and thus amplified upconverted emissions.^163^ Increase of the upconversion efficiency in energy migration‐mediated core and shell structured UCNPs is an appealing direction.

### 6.2 Ultralow‐Intensity NIR‐Activated Drug Release

The incorporation of photoresponsive materials with red‐shifted absorptions that is a better match with the anti‐Stokes emissions of the UCNPs will also improve the photoreaction efficacy of NIR‐initiated drug release, thus eliminating the need for high intensity NIR illumination. The photon numbers for excitation of different upconverted emissions from UCNPs differ and according to the intensity‐power curves of a set of UCNPs, upconverted spectrum at 340 nm can be attributed to a five‐photon process, emission at 360 nm is a four‐photon process, while blue emission at 475 nm is a three‐photon pathway.^50^, ^52^, ^56^ Thus, anti‐Stokes blue emissions from UCNPs requires a lower power intensity from the photon source to exceed the threshold limit for enabling NIR‐to‐visible emission.^150^ Following this concept, Wu and co‐workers conducted a series of investigations to measure the excitation threshold limit required to facilitate UV and visible emissions from the most frequently used NaYF_4_:Yb/Tm@NaYF_4_ UCNPs. Both UV and blue lights emitted upon high 974 nm excitation intensities (≥5.48 W cm^–2^). However, at lower excitation intensities (0.19–0.81 W cm^–2^), emission in the UV range (<400 nm) vanished completely while blue emissions (470 nm) were still emitted.^165^

**Figure 9:**
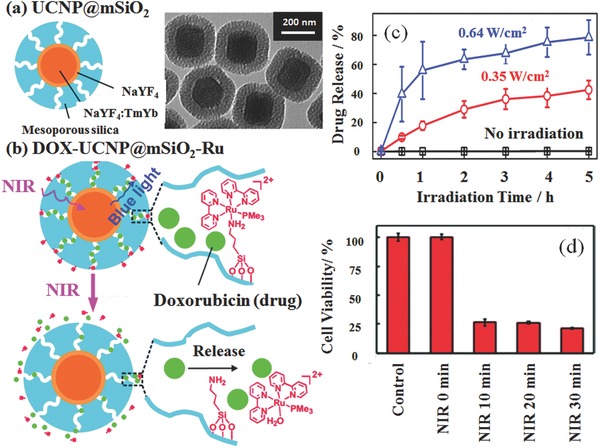
a) Schematic model and TEM image of UCNP@mSiO_2_ nanoparticles. Schematic illustration: upconverted blue luminescence triggers cleavage of Ru complexes and release of doxorubicin from DOX‐UCNP@mSiO_2_‐Ru nanoparticles. b) Doxorubicin release profile for PEG‐ and FA‐modified DOX‐UCNP@mSiO_2_‐Ru nanoparticles in the dark and upon 974 nm light exposure. The release profile was measured by fluorescence spectroscopy. c) Viability of HeLa cells when a 1 mm thick pork tissue is placed between 974 nm light (0.64 Wcm^–2^) and PEG‐and FA‐modified DOX‐UCNP@mSiO_2_‐Ru nanoparticles. Reproduced with permission.^165^ Copyright 2015, Royal Society of Chemistry.

To this end, Wu's group for the first time incorporated blue‐light‐cleavable ruthenium (Ru) complexes into the surface of UCNPs for drug release initiation. Through using ultralow‐intensity 974 nm photon sources at 0.35 W cm^–2^, upconverted blue emissions photocleaved the Ru complex, which ultimately triggered the release of DOX from DOX‐UCNP@mSiO_2_‐Ru (**Figure** **9**). In their UCNPs‐assisted DDSs, 42% and 78% of DOX was released after 974 nm illumination (5 h) at 0.35 W cm^–2^ and 0.64 W cm^–2^ respectively. Interestingly, only ≈27% of DOX was released from UV‐cleavable azobenzene–grafted UCNPs under identical conditions (947 nm illumination for 5 hr) with much higher power intensity of 7 W cm^–2^. Furthermore, in cellular studies, phototrigger DOX‐release decreased Hela cells viability to a value of 40–29% after 974 nm radiation (0.64 W cm^–2^) for 10–30 min. Upon exposure of water to 0.35 W cm^–2^ NIR light, there is minimal temperature increase of only 2.5 °C which minimized the overheating problems and prevented photodamage to the biological system.^165^

**Figure 10:**
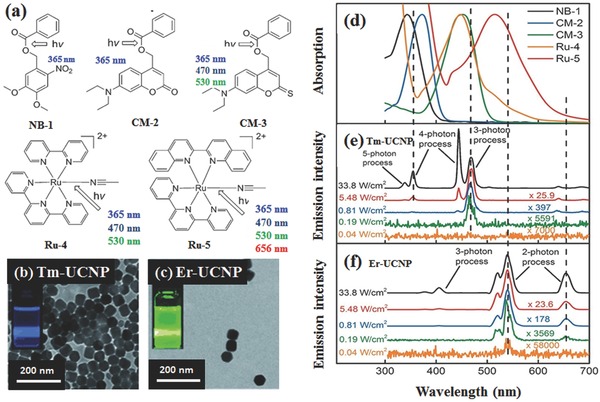
a) Chemical structures of five photocleavable compounds. The photocleavable bonds are indicated by the arrows. b,c) TEM images of Tm‐UCNP and Er‐UCNP. The insets show photographs of the UCNPs after *λ* = 974 nm laser exposure d) UV/Vis absorption spectra of five photocleavable compounds. e,f) Emission spectra of Tm‐UCNP and Er‐UCNP excited by a *λ* = 974 nm laser with different excitation intensities. The excitation intensity was controlled by a laser driver. The emission intensities of Tm‐UCNP and Er‐UCNP are normalized at *λ* = 470 and 540 nm, respectively. Reproduced with permission.^166^

In another study carried out by the same group, upconverted emissions from two diverse Yb^3+^‐sensitized UCNPs doped with Tm and Er (Tm‐UCNP and Er‐UCNP) were investigated upon NIR light irradiation with different power intensities. Additionally, absorption spectra of five various photoresponsive compounds (nitrobenzyl derivative: NB‐1, two types of coumarin derivative: CM‐2 and CM‐3; and two types of Ru complex: Ru‐4 and Ru‐5) were evaluated (**Figure** **10**a,d) in order to design an appropriate combinations of UCNPs and photocleavable materials to lessen the excitation thresholds required for photoreaction activation.^57^ Photon upconversion studies on the Tm‐UCNPs revealed that the excitation thresholds for the ^1^I_6_–^3^F_4_ transition (*λ* = 340 nm, five‐photon process^56^, ^57^), the ^1^D_2_–^3^H_6_ transition (*λ* = 360 nm, four‐photon process^56^, ^57^), the ^1^D_2_–^3^F_4_ transition (*λ* = 450 nm, four‐photon process^56^, ^57^), and the ^1^G_4_–^3^H_6_ transition (*λ* = 475 nm, three‐photon process^56^, ^57^) were 5.5, 2.2, 0.64, and 0.19 W cm^–2^, respectively (Figure 10e). While based on the Er‐UCNP upconverted emissions, the excitation thresholds for the ^2^H_9/2_–^4^I_15/2_ transition (*λ* = 409 nm, three‐photon process^56^), the ^2^H_11/2_–^4^I_15/2_ transition (*λ* = 520 nm, two‐photon process^56^), the ^4^S_3/2_–^4^I_15/2_ (*λ* = 540 nm, two‐photon process^56^), and the ^4^F_9/2_–^4^I_15/2_ transition (*λ* = 653 nm, two‐photon process^56^) were 2.2, 0.09, 0.04, and 0.09 W cm^–2^, respectively (Figure 10f). Therefore, upon Tm‐UCNPs excitation, the blue emission remains at low power intensities (0.19–2.2 W cm^–2^) while the UV emission vanishes completely. Also upon low excitation intensity (0.09–2.2 W cm^–2^) of Er‐UCNPs, only upconverted green and red emissions remain and blue emission disappears entirely.

**Figure 11:**
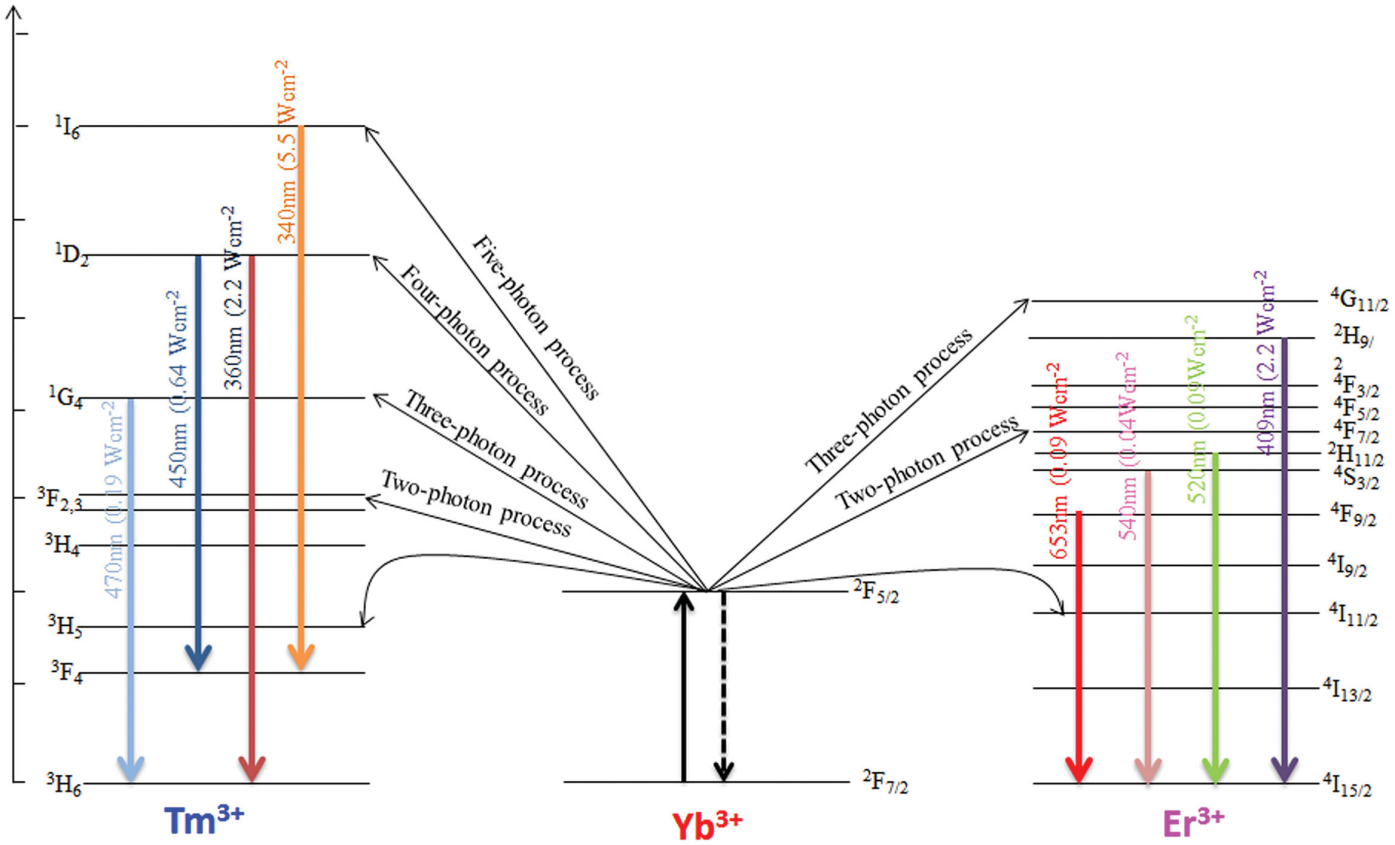
Excitation threshold required for different photon pathways based on the upconverted emissions with different wavelength. Data obtained from refs.^43^, ^52^, ^57^, ^165^, ^166^

**Figure** **11** summarized the excitation threshold required for different photon pathways of Tm and Yb dopants based on the upconverted emissions with different wavelength. Amongst the studied photoresponsive materials only CM‐3, Ru‐4, and Ru‐5 can be induced by either the four‐photon NIR‐to‐UV pathway (higher excitation intensity) or the three‐photon NIR‐to‐blue pathway (lower excitation intensity). Consequently, using Tm‐UCNPs as an internal blue light source upon low NIR excitation intensities facilitates the photocleavage of CM‐3, Ru‐4, and Ru‐5. Interestingly, cleavage of Ru‐4 and Ru‐5 can be initiated by the two‐photon NIR‐to‐green process which can be provided by Er‐UCNPs.^166^ According to the reported excitation threshold of upconverted emissions, visible‐light‐sensitive materials like Ru complexes are suitable for NIR‐initiated DDSs due to the less photodamage to biomaterials. In general, combining visible‐light sensitive compounds with UCNPs which could efficiently reduce the excitation intensity to initiate the UCNPs‐assisted photoreaction is an ultimate objective of researchers to reach a medically safe level for advancing the biomedical applications of Yb^3+^‐sensitized UCNPs‐based carrier systems.

### 6.3 Tuning the UCNPs Excitation Wavelength

#### 6.3.1 Nd^3+^‐Sensitized UCNPs

**Figure 12:**
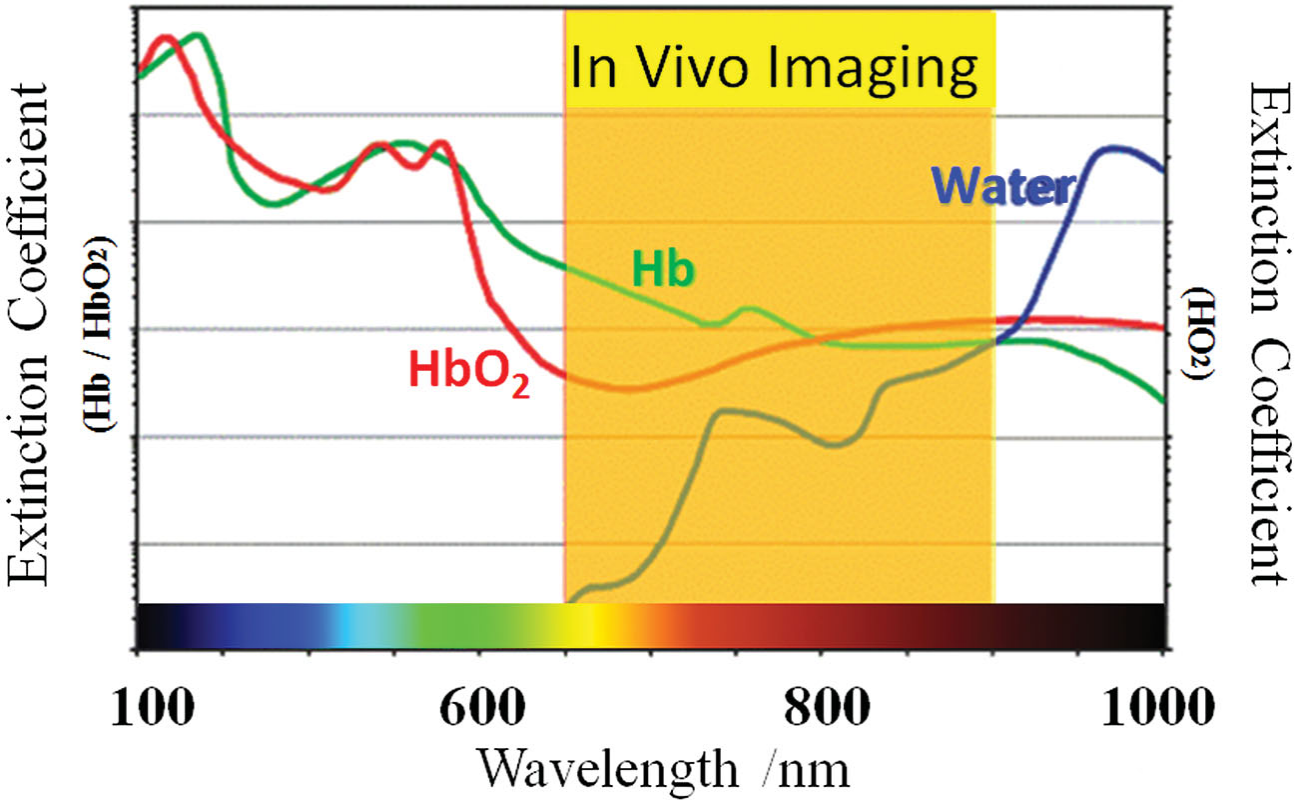
Spectra profiles of tissue optical window. The extinction coefficient of water at 800 nm is about 20 timers lower than that at 980 nm (Hb: hemoglobin; HbO 2: oxyhemoglobin). Reproduced with permission.^167^

Tuning the UCNPs excitation wavelength is of considerable practical significance for the expansion of new generations of UCNPs with substitute excitation wavelength for in vitro and in vivo studies. Until now, few laboratories have concentrated on utilizing light of other wavelengths to excite UCNPs. He's group replaced the 980 nm source with a 915 nm laser illumination to excite Yb^3+^‐sensitized NaYF_4_: Ln nanoparticles, which showed less heating of the biological sample and enhanced deep tissue imaging capability due to relatively low water absorption.^141^ Nonetheless, this excitation wavelength still partially overlaps with the absorption peak of water. Therefore, ongoing efforts have been devoted toward preparing new series of UCNPs that can be excited by ≈800 nm illumination where water has the local minima absorption coefficient while it is still in the tissue optical window (**Figure** **12**).^167^, ^168^, ^169^, ^170^, ^171^ In this regard, in 2013, Han and co‐workers designed the first generation of Nd/Yb/Er (Tm) tri‐doped cascade sensitized core/shell UCNPs with the ability to be excited by 800 nm laser. In their engineered UCNPs system, small amount of Nd^3+^ (doping ratio into the core of ≤1%, undoped shell) played a vital role in sensitizing the UCNP owing to the extreme absorption cross‐section at ≈800 nm and competent energy transfer between Nd^3+^ and Yb^3+^. Based on their findings, all three lanthanides (Nd/Yb/Er (Tm)) are essential for the proposed cascade sensitization mechanism. Nd^3+^ acts as sensitizer and absorbs photons while Yb^3+^ participate as bridging ions in order to transfer energy from Nd^3+^ to the emitters Er^3+^/Tm^3+^ (Figure 1b).^167^ Their new class of UCNPs outperformed conventional 980 nm‐excited Yb^3+^‐sensitized dual‐doped UCNPs in regard to considerably reduced NIR photon absorption and thus decreased deleterious heating effects while enhancing penetrability through water.^167^ However, Han et al.'s design, low concentration of Nd^3+^ was doped within the core structure (with optimal doping ratio of 0.5% inside core of Er^3+^‐UCNPs and 1% inside core of Tm^3+^‐UCNPs) to avoid deleterious cross‐relaxation routes while resulting in weak absorption at 800 nm and eventually weak anti‐Stock emissions.

**Figure 13:**
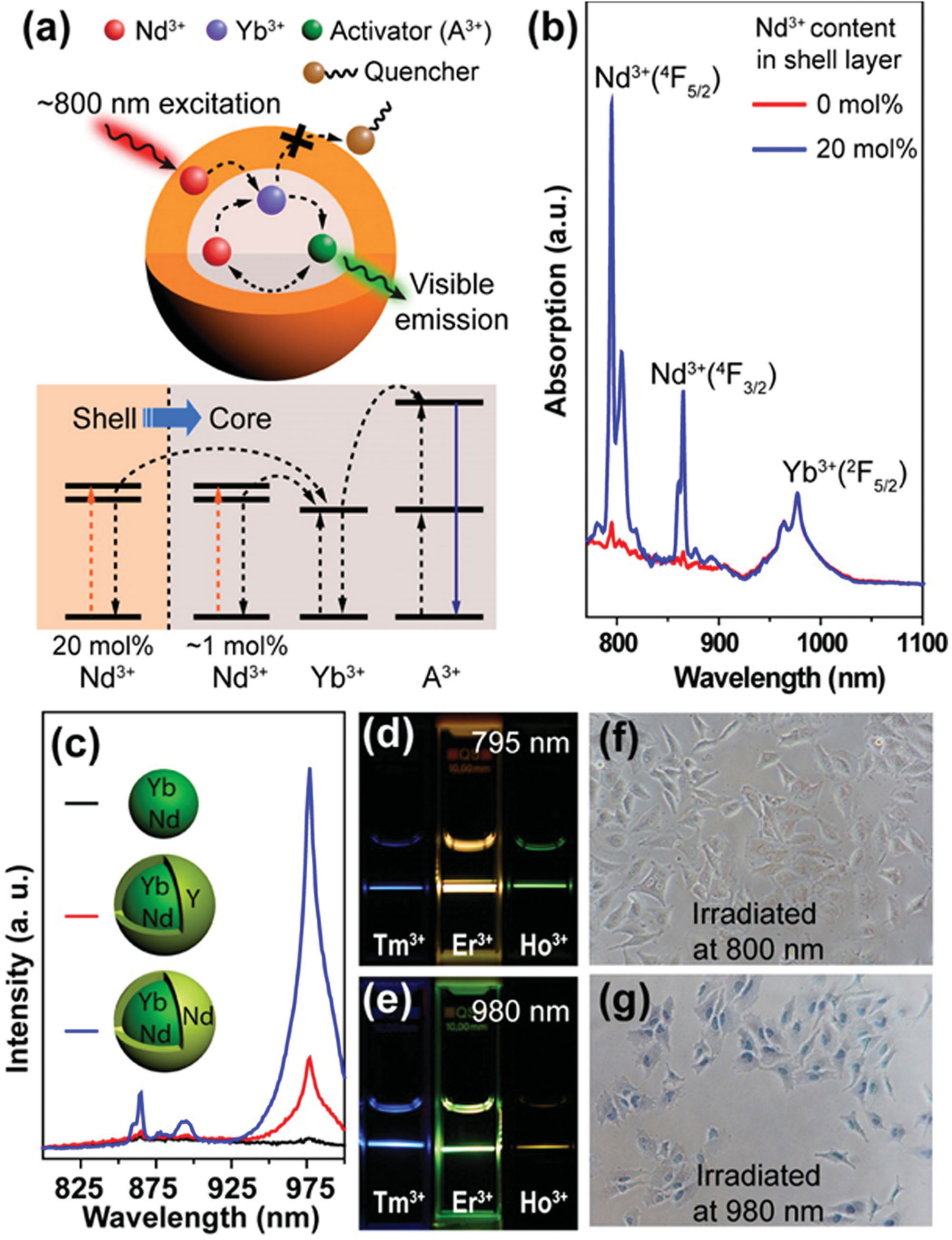
a) Schematic design (top) and simplified energy level diagram (bottom) of a core−shell nanoparticle for photon upconversion under 800 nm excitation. Nd^3+^ ions doped in the core and shell layers serve as sensitizers to absorb the excitation energy and subsequently transfer it to Yb^3+^ ions. After energy migration from the Yb^3+^ ions to activator ions, activator emission is achieved via the Nd^3+−^sensitization process. b) Near‐IR absorption spectra of NaYF_4_:Yb/Nd (30/1%) nanoparticles coated with an inert NaYF_4_ shell or an active NaYF_4_:Nd (20%) shell. The absorption spectra were normalized at 976 nm for comparison. c) Near‐IR photoluminescence spectra of NaYF_4_:Yb/Nd (30/1%) nanoparticles and the corresponding core−shell nanoparticles coated with NaYF_4_ or NaYF_4_:Nd(20%). d,e) Luminescence photographs of activator emissions (Tm 0.5%, Er 0.5%, Ho 1%) for Nd^3+^‐sensitized and Yb^3+^‐sensitized nanoparticles under 795 and 980 nm irradiation, respectively (laser power: 100 mW). The same particle concentration for each set of comparisons was obtained by normalizing the particle absorption at 976 nm. f, g) Optical microscopy images of trypan blue‐treated HeLa cells recorded after irradiation for 5 min at 800 and 980 nm, respectively (6 W cm^–2^). Reproduced with permission.^61^ Copyright 2013, American Chemical Society.

Liu's group formulated a new class of core‐shell UCNPs with high‐doping concentration of Nd^3+^ (≈20 mol%) within the shell layer for effective absorbance of 800 nm light, and a relatively low concentration of Nd^3+^ (1–2mol %) doped inside the core to prevent detrimental cross‐relaxation route between Nd^3+^ and emitters (**Figure** **13**a).^61^ Remarkably, NIR absorption spectra of nanoparticles with an active NaYF_4_:Nd(20%) shell was almost 17 times stronger compared with nanoparticles treated with an inert NaYF_4_ shell (Figure 13b–e).^61^ Comparison between cell viabilities of Hela cells upon absorbing 800 and 980 nm light (under identical conditions: 5 min illumination at 6 W cm^–2^) showed that the cancer cells remained intact after 800 nm irradiation while almost all the cancer cells were killed with 980 nm illumination (Figure 13f,g).

**Figure 14:**
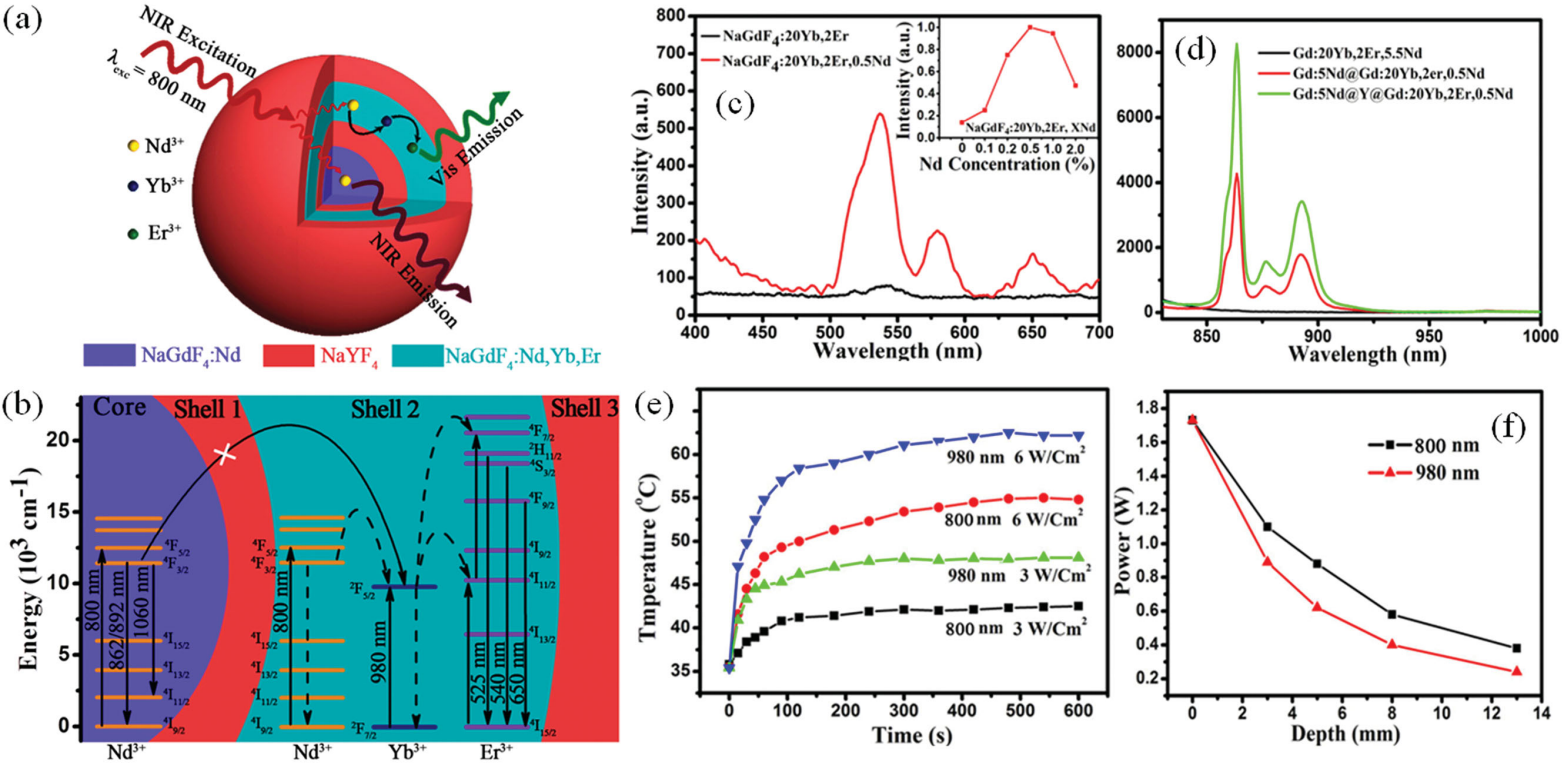
a) General strategy to achieve the UC and DC dual‐mode luminescence with core/shell_1_/shell_2_/shell_3_ (β‐NGdF_4_: Nd/NaYF_4_/NaGdF_4_: Nd, Yb, Er/NaYF_4_). b) Proposed energy transfer mechanisms in the multi‐layer core/shell NCs. c) UC emission spectra of the NaGdF_4_:20Yb, 2 Er and NaGdF_4_:0.5Nd, 20 Yb, 2 Er under 800‐nm excitation. The inset shows the UC emission intensity of the NaGdF_4_: Nd, Yb, Er NCs as a function of different Nd^3+^ ion doping concentrations under 800‐nm excitation. d) DC emission spectra of NaGdF_4_:Nd,Yb,Er,NaGdF4:Nd/NaGdF4:Nd,Yb,Er, and NaGdF4:Nd/NaYF4/NaGdF_4_:Nd,Yb Er NCs under 800‐nm excitation. e) Time‐resolved temperature in the irradiated nude mouse skins during 10 min irradiation of 980‐ and 800‐nm laser as a function of different power density. f) The decay of excitation power as a function of the penetration depth of tissues. Reproduced with permission.^51^ Copyright 2013, Nature Publishing Group.

Likewise, Yan and co‐workers extended excitation bands to shorter wavelengths (808 nm) by introducing Nd^3+^ to core/shell UCNPs which not only showed high upconversion emissions comparable to that excited at 980 nm but also minimized laser‐generated overheating issues attributed to Yb^3+^‐based UCNPs.^172^ Another striking example was reported by Zhao's group who designed a multi‐layer core/shell_1_/shell_2_/shell_3_ (β‐NGdF_4_: Nd/NaYF_4_/NaGdF_4_:Nd,Yb,Er/NaYF_4_) UCNPs with an efficient 800 nm excitable exclusivity. This unique engineered system opened a new avenue towards new classes of nanocrystals with dual‐mode upconverting (800–540 nm) and downconverting (800 to 860–895 nm) ability for in vitro and in vivo bioimaging (**Figure** **14**a–d). Heat issues and penetration depth of the nanoparticles were investigated and compared with 980 nm excitable Yb^3+^‐sensitized UCNPs which showed lower heat increase and higher penetration depth (Figure 14e,f).^51^

**Figure 15:**
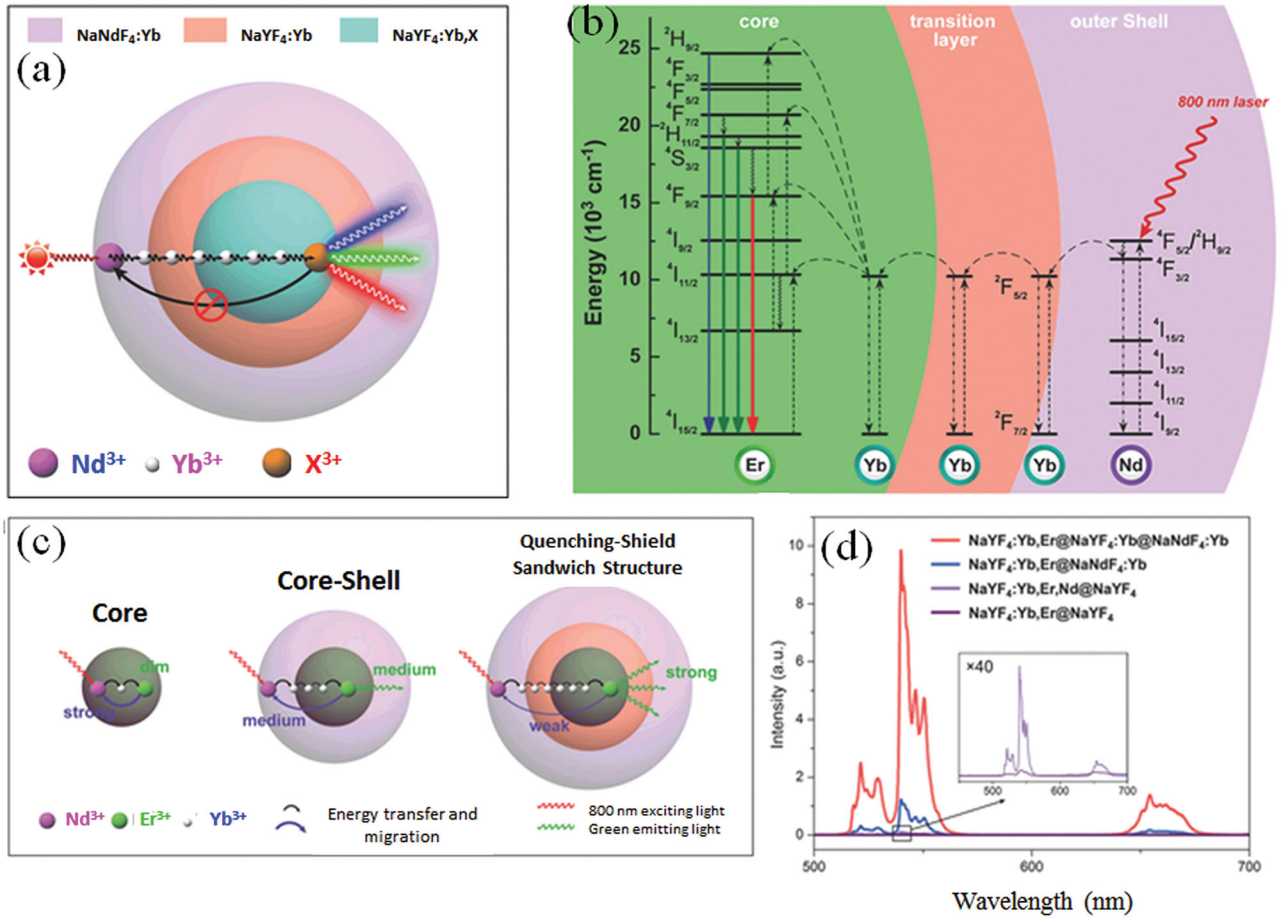
a) Schematic illustration of the proposed energy‐transfer mechanisms in the quenching‐shield sandwich‐structured UCNPs upon 800 nm excitation. b) Proposed energy‐transfer mechanisms in the quenching‐shield sandwich nanoparticle upon 800 nm diode‐laser excitation. Regulating the energy transfer and migration in quenching‐shield sandwich nanostructures to gain bright UC luminescence upon excitation at 800 nm. c) Schematic illustration of the energy‐transfer and migration processes in the Nd^3+^ ‐sensitized triply doped, NaNdF_4_ ‐coated core‐shell, and quenching‐shield sandwich nanoparticles. d) UC emission spectra of the synthesized quenching‐shield sandwich nanoparticles (red line), the Nd‐coating core‐shell nanoparticles (blue line), the Nd/Yb/Er triply doped nanoparticles (violet line), and the conventional Yb^3+^ ‐sensitized UCNPs (dark violet line) under 800 nm excitation (0.5 W cm^–2^). All of the NPs were hexagonal phase and dispersed in cyclohexane at the same concentration (20 mg mL^–1^). Reproduced with permission.^55^

Elimination of cross‐relaxation effects was highly desirable for Nd^3+^‐doped UCNPs. Yao and co‐workers designed a distinctive quenching‐shield sandwich nanostructure in order to completely eliminate the deleterious energy transfer (photon quenching effect initiated by energy back transfer from activators to ^4^I_J_ manifolds of Nd^3+^) between activators and sensitizer. In their approach, a transition layer of NaYF_4_ was introduced between the core (Er^3+^ was doped) and outermost shell (Nd^3+^ was embedded in). This suppressed the detrimental cross‐relaxation pathways between activator and sensitizers and eventually achieved the maximum harvest of 800 nm light. The results not only indicated 125 times stronger upconverted luminescence from Nd^3+^‐doped UCNPs compared with conventional 980 nm‐excited UCNPs but also minimized overheating issues and resulted in deeper tissue penetration (**Figure** **15**).^55^ Lin and co‐workers extended usage of 808 nm excited Nd^3+^‐doped UCNPs to successfully target cancer tumor; this is an important step towards anti‐cancer chemotherapy and biological imaging applications without detrimental overheating problems.^173^

#### 6.3.2 Dye‐Sensitized UCNPs

Although doping Nd^3+^ ions into the UCNPs generated a bonus excitation peak at around 800 nm, the fundamental limitation of UCNPs still remains which is the weak and narrowband absorption of lanthanide sensitizers (Nd^3+^ and Yb^3+^). This major limitation that stems from the nature of the 4f–4f electronic transitions of these ions hinders broad harvesting of NIR light by UCNPs, leading to low upconverting quantum efficiency. Recently, NIR dye‐sensitized UCNPs have emerged as an appealing alternative for classical UCNPs in order to enhance the upconverting efficiency.^38^, ^174^ The idea of using organic NIR dye (acting as an external antenna) for UCNPs to increase the absorptivity and total broadening of the absorption spectrum range of the nanoparticles was first presented by Hummelen's group.^175^ Although their unique dye‐sensitized upconverting process dramatically enhanced the upconversion efficiency, poor overlap between dye emission and nanocrystal absorption resulted in an inefficient energy transfer between them. Furthermore, β‐NaYF_4_:Yb Er UCNPs (without shell) was used in their design in order to allow dye sensitization leading to intense surface‐related upconverting emission quenching (upconversion quantum efficiency of ≈0.2%).^175^ To address this problem, Chen et al. introduced and validated a new concept of multistep energy‐cascaded upconverting process by combining the merits of core@shell structured UCNPs and NIR dyes with overlapping absorption spectrum.^38^ In their design, various NIR dyes were anchored on the surface of the core@shell structured UCNPs (NaYbF_4_:Tm 0.5%@NaYF_4_:Nd 30%) with a hierarchical alignment of energy levels (the NIR dye, the intermediate sensitizer Nd^3+^, and the sensitizer Yb^3+^) to broadly harvest NIR light and initiate the unidirectional energy transfer of the harvested energy to the lanthanide activators located within the core of the nanoplatform. Additionally, due to the usage of core@shell structure, surface‐related deactivation and upconverted emission quenching was suppressed within the shell layers. The harvested energy by dyes was nonradiatively transferred to the sensitizer Nd^3+^ ions (82% efficiency) and, sequentially, to the Yb^3+^ ions (80% efficiency) which sensitized Tm^3+^ ions within the core. These dye‐sensitized core and shell UCNPs facilitated three‐, four‐ and five‐ photon upconversion quantum efficiency as high as 19% (upconversion energy conversion efficiency of 9.3%, upconversion quantum yield of 4.8%) upon exposure to a broad NIR spectral range of 700 to 850 nm. This energy‐cascaded upconverting process was proven to be effective using different types of organic dyes, activators and fluoride host lattices. Interestingly, co‐sensitization of core@shell UCNPs by commercially available IR‐808 and IR‐820 dyes, generated unprecedented anti‐Stokes blue emissions (visible by naked eyes) through a three‐photon process in response to 750–1100 nm spectral range illumination at 0.016 W cm^–2^ power intensity.^38^

In another study, Han's group demonstrated an innovative dye‐sensitized core and shell UCNPs (IR‐806‐β‐NaYF_4_:20%Yb,2%Er@*β*‐NaYF_4_:10%Yb) with enhanced absolute quantum yield of ≈5% under 800 nm excitation (2 W cm^–2^), which was significantly higher than the current highest value (0.18% at 31 W cm^–2^)^173^ reported for existing 808 nm excitable UCNPs (NaYF_4_:Yb,Er@NaYF_4_:Yb@NaNdF_4_:Yb@NaYF_4_:Yb).^176^ Moreover, their engineered dye‐sensitized UCNPs showed a broadened absorption range with enhanced ≈20‐fold integrated spectral response upon exposure to the wavelength range 720–1000 nm in comparison with the same UCNPs without dye sensitizing. These nanoarchitectures were also utilized for optogenetics and bioimaging applications.^176^ Using dye‐sensitized core and shell UCNPs have shown promise in amplifying the upconversion efficiency and broadening the absorption range, which will pave the way for new biomedical application of UCNPs and particularly for NIR‐triggered controlled release of therapeutic compounds.

## 7 Conclusion and Future Prospects

Photocontrollable UCNPs has offered a high degree of spatial and temporal determination for drug delivery processes using NIR light, an external stimulus that is highly compatible with biological systems. NIR‐initiated UCNPs‐based DDSs have the potential to overcome the problems associated with conventional light‐activated DDSs, including enhanced biological tissue penetration depths realized by the NIR excitation and less photo induced damage to live tissues caused by high energy excitation source.

Several different strategies have been developed to integrate various functional moieties with the UCNPs to form NIR‐initiated DDSs, including the incorporation of inorganic material such as silica or polymers to improve the dispersion of the UCNPs in an aqueous environment and for subsequent conjugation with drug and targeting moieties. NIR light‐activated drug release can be achieved with the use of photocleavable or ‘‘caged’’ molecules, and with photoswitching of molecules. UCNP‐based nanocomposites have been exploited as targeted DDSs by using a range of bio‐recognition molecules, such as antibodies, peptides and FA or the incorporation of magnetic material into the UCNP‐based carrier systems.

Nonetheless, despite the rapid development of UCNPs‐based DDSs in the last five years, several technical challenges remain, which hinder their practical applications. Firstly, since the majority of photoresponsive materials require UV light to initiate the photoreaction, the effectiveness of NIR‐initiated DDSs is greatly dependant on the intensity of upconverted UV light which is correlated to the excitation intensity of NIR photon source. On the other hand, absolute quantum yield of the developed UCNPs is relatively low which arouse the need for high power excitation. Therefore, the development of highly efficient NIR‐to‐UV nanoparticles with enhanced quantum yields (with no heating issues) will be of considerable practical importance. Secondly, introducing photoresponsive compound with red‐shifted absorption that eliminates the need for high intensity excitation is greatly demanded. Nonetheless, the potential long term toxicity and stability of photoresponsive materials and lanthanide dopants needs more advanced evaluation for in vivo applications. Thirdly, most of the apparatus employed for optical characterization of these nanoparticles are laboratory customized and all the commercial instruments are typically designed with down‐conversion probes. Accordingly, ready‐to‐use and standard instruments are greatly required to facilitate accurate quantitative and comparable measurements. Fourthly, dye‐sensitized UCNPs as well as Nd^3+^‐sensitized UCNPs though promising as more efficient systems (compare with classical Yb^3+^‐sensitized one) with greater upconversion quantum efficiency, biocompatibility, minimized overheating effects and deeper penetration depth still face certain challenges. Designing and optimizing the core‐shell structure of these nanoplatforms is becoming more and more complex, indicating that current synthesis approaches hardly actualize the clinical needs. Thus, the development of new synthesis technique is in great need. Taken all together, this exciting field is developing at a very fast pace and there are plenty of scope for further innovative studies. This extremely active research area requires multidisciplinary collaborative research and with more focus it is expected to open new promises in nanomedicine.
